# RIPK3 controls MAIT cell accumulation during development but not during infection

**DOI:** 10.1038/s41419-023-05619-0

**Published:** 2023-02-11

**Authors:** Timothy Patton, Zhe Zhao, Xin Yi Lim, Eleanor Eddy, Huimeng Wang, Adam G. Nelson, Bronte Ennis, Sidonia B. G. Eckle, Michael N. T. Souter, Troi J. Pediongco, Hui-Fern Koay, Jian-Guo Zhang, Tirta M. Djajawi, Cynthia Louis, Najoua Lalaoui, Nicolas Jacquelot, Andrew M. Lew, Daniel G. Pellicci, James McCluskey, Yifan Zhan, Zhenjun Chen, Kate E. Lawlor, Alexandra J. Corbett

**Affiliations:** 1 Department of Immunology and Microbiology, University of Melbourne at the Peter Doherty Institute for Infection and Immunity, Melbourne, Victoria Australia; 2 Centre for Innate Immunity and Infectious Diseases, Hudson Institute of Medical Research, Clayton, Victoria Australia; 3 State Key Laboratory of Respiratory Disease, Guangzhou Institute of Respiratory Disease, Guangzhou Medical University, Guangzhou, China; 4 Walter and Eliza Hall Institute of Medical Research, Parkville, Victoria Australia; 5 Department of Medical Biology, University of Melbourne, Parkville, Victoria Australia; 6 Department of Molecular and Translational Science, Monash University, Clayton, Victoria Australia; 7 Murdoch Children’s Research Institute, Royal Children’s Hospital Parkville, Parkville, Victoria Australia; 8 Department of Paediatrics, University of Melbourne, Parkville, Victoria Australia; 9 Department of Drug Discovery, Shanghai Huaota Biopharm, Shanghai, China

**Keywords:** Cell death and immune response, Mucosal immunology, Immune cell death

## Abstract

Cell death mechanisms in T lymphocytes vary according to their developmental stage, cell subset and activation status. The cell death control mechanisms of mucosal-associated invariant T (MAIT) cells, a specialized T cell population, are largely unknown. Here we report that MAIT cells express key necroptotic machinery; receptor-interacting protein kinase 3 (RIPK3) and mixed lineage kinase domain-like (MLKL) protein, in abundance. Despite this, we discovered that the loss of RIPK3, but not necroptotic effector MLKL or apoptotic caspase-8, specifically increased MAIT cell abundance at steady-state in the thymus, spleen, liver and lungs, in a cell-intrinsic manner. In contrast, over the course of infection with *Francisella tularensis*, RIPK3 deficiency did not impact the magnitude of the expansion nor contraction of MAIT cell pools. These findings suggest that, distinct from conventional T cells, the accumulation of MAIT cells is restrained by RIPK3 signalling, likely prior to thymic egress, in a manner independent of canonical apoptotic and necroptotic cell death pathways.

## Introduction

T cells encompass a broad range of effector, memory and regulatory subpopulations; each defined by distinct phenotypic characteristics and effector functions. While T cells play a fundamental role in mediating protection against infection and cancer, their responses need to be tightly regulated to prevent excessive inflammation and damage to healthy tissue. For example, following an infection, cell death is a key process to reduce the population size of immune cells and allow resolution of tissue homeostasis [1].

Mucosal-associated invariant T (MAIT) cells are innate-like T cells that, in humans, are relatively abundant in the circulation and at mucosal sites, such as the lungs and in the liver (up to 40% of αβ T cells) [2, 3]. In mice, MAIT cells have a similar distribution to that in humans, but are less abundant, representing, for example, 0.5-2% of αβ T cells in the lungs [4–6**]**. Unlike conventional T cells, which recognise antigenic peptides presented by class I or class II MHC proteins, MAIT cells recognise antigens presented on the MHC-I related protein 1 (MR1) though their semi-invariant T cell receptor (TCR). To date, the most potent and best studied MAIT cell antigen is 5-(2-oxopropylideneamino)-6-D-ribitylaminouracil, 5-OP-RU), which is derived from microbial riboflavin (vitamin B2) biosynthesis metabolites [7]. During bacterial infections, MAIT cells rapidly expand within tissues and may comprise up to 50% of αβ-T cells in infected organs [5, 8, 9]. MAIT cells have been shown, largely via studies using MAIT cell-deficient *Mr1*^−/−^ mice, to be important for control of several bacterial pathogens, including *Legionella longbeachae* [8], *Mycobacterium bovis* BCG [10] and *Francisella tularensis* live vaccine strain (LVS) [11, 12]. Moreover, MAIT cells expanded by antigen priming can enhance protection from several pathogens [8]. However, MAIT cells can also become dysregulated and drive unwanted self-destructive immune responses to chronic infections in mice [13] and in humans [14]. Thus, understanding the mechanisms controlling the development, proliferation, functional activation, and survival of MAIT cells may help in the design of immune interventions to maximize the beneficial impact of MAIT cells while curtailing their potentially pathogenic effects.

Three major forms of programmed cell death have been implicated in conventional T cell turnover - mitochondrial “intrinsic” apoptosis, death receptor-induced “extrinsic” apoptosis and necroptosis [15]. Mitochondrial apoptosis is an immunologically silent form of cell death triggered by cell stress. In contrast, death receptor-induced apoptosis is triggered upon death receptor ligation and subsequent formation of a death inducing signalling complex [16]. In the case of TNFR1 ligation by TNF, this complex essentially comprises RIPK1-FADD-caspase-8, although a RIPK1-RIPK3-FADD-caspase-8 ripoptosome complex can also form under certain conditions in select cells [e.g., inhibition of the inhibitor of apoptosis proteins (IAPs) using small molecule SMAC mimetics] [17**–**19]. When caspase-8 activity is inhibited, a form of lytic cell death, termed necroptosis, can be triggered, which is dependent on the kinase activities of RIPK1 and/or RIPK3, and the RIPK3 substrate, pore forming protein MLKL [15, 20].

Clearance of activated conventional T cells has been linked to death receptor-induced apoptosis, depending on the experimental conditions, such as the pathogenic insult they are exposed to (reviewed in [15, 21, 22]). Activated, but not naïve, T cells are also subject to necroptosis when caspase-8 activity is absent or inhibited [23**–**25]. This difference may be explained by the distinct MLKL expression patterns in T cells, specifically, MLKL expression is low in naïve T cells but is inducible upon activation [25, 26]. In contrast to conventional T cells, much less is known about MAIT cell regulation, including cell death-mediated regulatory events. However, MAIT cells have been previously reported to exhibit a greater propensity for apoptosis than conventional T cells, due to increased capsase-3 and -7 activity under the control of the innate cell restricted transcription factor PLZF [27]. Intriguingly, both mouse and human MAIT cells differ from conventional T cells in that they display an ‘effector memory’ phenotype on thymic egress. Namely, MAIT cells express high levels of CD44 and low levels of CD62L, even in the absence of pathogenic or antigenic insult. MAIT cells are further activated and expand following infection, resulting in a long-lasting memory-like population [8, 12]. It therefore remains likely that the regulation of the cell death program in MAIT cells is distinct from that of conventional T cells.

In this study, we investigated the molecular requirements for MAIT cell survival basally and during infection in mice. Interrogation of molecules that regulate extrinsic cell death revealed that MAIT cells differ greatly from conventional T cells, as they basally expressed necroptotic machinery RIPK1, RIPK3 and MLKL. Importantly, we found that RIPK3 signalling, selectively and cell-intrinsically, repressed MAIT cell accumulation in lymphoid organs and peripheral tissues. Surprisingly, this RIPK3-mediated effect did not appear to be dependent on downstream apoptotic caspase-8 or necroptotic MLKL activity. Collectively, our study suggests a novel role for RIPK3 in regulating MAIT cell abundance at steady state.

## Results

### RIPK3 deficiency results in a preferential increase in MAIT cell abundance

**Fig. 1:**
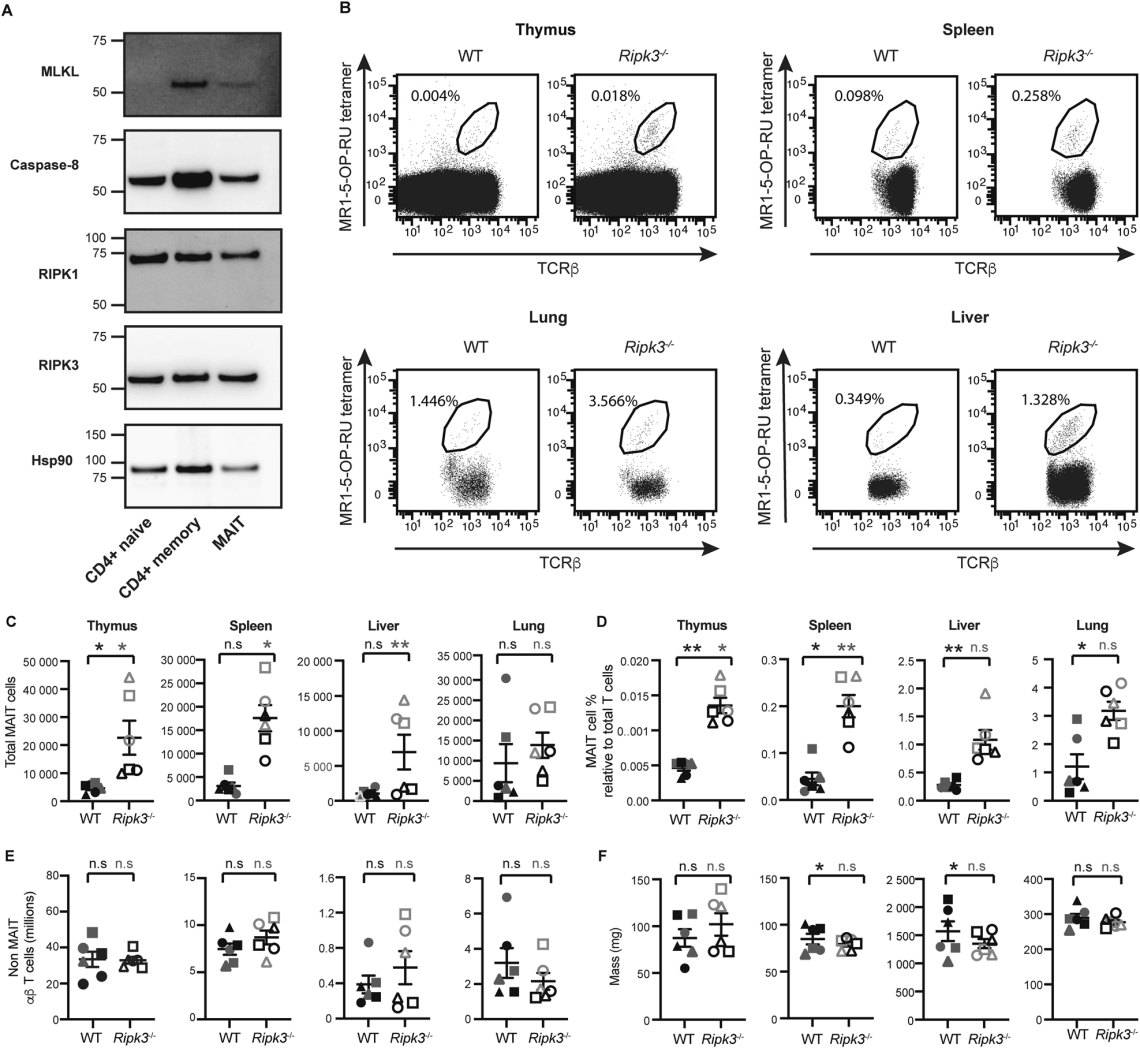
RIPK3 deficiency selectively affects MAIT cell abundance. **A** Western blot showing presence of the indicated molecules in lysates of FACS-sorted liver MAIT cells, naïve (CD62L^+^, CD44^−^) CD4^+^ T cells, and memory (CD62L^−^, CD44^+^) CD4^+^ T cells at steady state. Data shown from 1 experiment representative of three independent experiments (*n* = 3). **B** Flow plots showing whole thymocytes, or αβ-T cells from spleen, liver and lungs of C57BL/6 (WT) and *Ripk3*^−/−^ mice. MAIT cell abundance is expressed as a percentage relative to non-MAIT TCRβ^+^ lymphocytes. **C** Absolute MAIT cell numbers, and **D** relative MAIT cell abundance showing MAIT cell % relative to total thymocytes for the thymus, and MAIT cell % relative to αβ T cells for all other organs (as in **B**), **E** absolute non-MAIT TCR β^+^ lymphocyte numbers in each of the indicated organs, and **F** mass of each organ. Data showing age-matched (~7.5 week old) male mice (black symbols) and female mice (grey symbols) for each WT (filled symbols) and *Ripk3*^−/−^ *mice* (hollow symbols), with each symbol (circle, square and triangle) representing an individual mouse across panels **C-F**. Bars show the mean ± SEM (*n* = 6 mice). Statistical significance is indicated for male (black annotations) and female (grey annotations) mice by ns (*p* > 0.05); * (*p* ≤ 0.05); ** (*p* ≤ 0.01); or *** (*p* ≤ 0.001) as determined by an unpaired two-tailed *t*-test with Welch’s correction for unequal variance. Data shown from a single experiment, representative of at least three independent experiments.

The survival mechanisms and modes of cell death that regulate conventional T cell pools have been relatively well defined. In contrast, little is known about cell death mechanisms operating in specialised innate-like T cells, including MAIT cells. To understand how regulation of MAIT cell death compares to that of conventional T cell subsets, we first examined the expression of key apoptotic and necroptotic signalling and effector proteins in sorted (Fig. S1) MAIT cells, CD4^+^ (or CD8^+^) naïve (CD62L^hi^ CD44^lo^) T cells and CD4^+^ memory (CD62L^lo^CD44^hi^) T cells from the liver (Fig. 1A) or spleen of naïve wild-type C57BL/6 mice (Fig. S2A) and C57BL/6 mice that had been treated with CpG + 5-OP-RU to increase MAIT cell numbers [12] by Western blot (Fig. S2B). We found that MAIT cells more closely resembled memory T cells as they expressed high levels of necroptotic effector MLKL (Fig. 1A and S2A). Fitting with a past report [15], MLKL was not detectable in naïve liver or splenic CD4^+^ T cells (Fig. 1A and S2A) or naïve splenic CD8^+^ T cells (Fig. S2A). MAIT cells also expressed the upstream kinases, RIPK1 and RIPK3 and apoptotic pro-caspase-8, at similar or higher levels than conventional naïve or memory T cells (Fig. 1A and Fig. S2A, B). These data suggest that, akin to conventional memory T cells, MAIT cells are competent to undergo RIPK1/3-dependent MLKL signalling. However, due to the low abundance of MAIT cells in the mouse (less than 0.1% of splenocytes), and the poor survival of murine T cells during in vitro culture, we have thus far been unable to directly test the capacity of murine MAIT cells to undergo necroptotic death.

RIPK3 can act as a dual positive regulator of extrinsic apoptotic and necroptotic cell death signalling [28]. We therefore next queried the role of RIPK3 in MAIT cell homeostasis. Enumeration of MAIT cells in RIPK3-deficient mice (Fig. 1B-F) revealed a marked increase in the absolute numbers of MAIT cells in the thymus, spleen, lungs and liver, when compared to wild-type (WT) controls (Fig. 1C). Importantly this difference translated to an increase in the relative abundance of MAIT cells in organs from both male and female *Ripk3*^−/−^ mice (Fig. 1D), apart from the liver and lungs in female *Ripk3*^−/−^ mice, which exhibited only a trend towards increased MAIT cell abundance (Fig. 1D). In contrast to the accumulation of MAIT cells in *Ripk3*^−/−^ mice, and consistent with past reports [29], we found no perturbation in the numbers of conventional αβ T cells in these mice (Fig. 1E). Nor did we find significant increases in the proportion of innate-like lymphoid cell populations that display both innate and adaptive features [8], including innate lymphoid cell (ILC) subsets in the lungs or small intestines (*i.e*., ILC1, ILC2 and ILC3; Fig. S3A, B) and NKT cells in the spleen, liver or lungs (Fig. S3C-F).

Considering the select increase in MAIT cell abundance in *Ripk3*^−/−^ mice across primary and secondary lymphoid compartments and peripheral sites, we postulated that these changes were caused by an increased thymic output of MAIT cells. Indeed, after normalising absolute MAIT cell counts per organ to that of the thymus, we found that there was no significant difference between MAIT cell peripheral expansion based on normalised abundance in the lungs, liver or spleen of *Ripk3*^−/−^ and WT mice (Fig. S3G). Collectively, these data suggest that RIPK3 has a unique role within the T cell compartment to regulate MAIT cell thymic output.

### Accumulation of MAIT cells in *Ripk3*^−/−^ mice occurs independent of caspase-8 and MLKL

Considering the function of the apoptotic initiator caspase, caspase-8, in the turnover of activated effector memory T cells [30], we next sought to determine the role of caspase-8 in limiting MAIT cell abundance. Given global caspase-8 deficiency unleashes lethal necroptotic cell death during embryogenesis [31], we examined MAIT cell numbers in mice genetically lacking both RIPK3 and caspase-8 (*Ripk3*^*−/−*^*Casp8*^*−/−*^ mice).

**Fig. 2:**
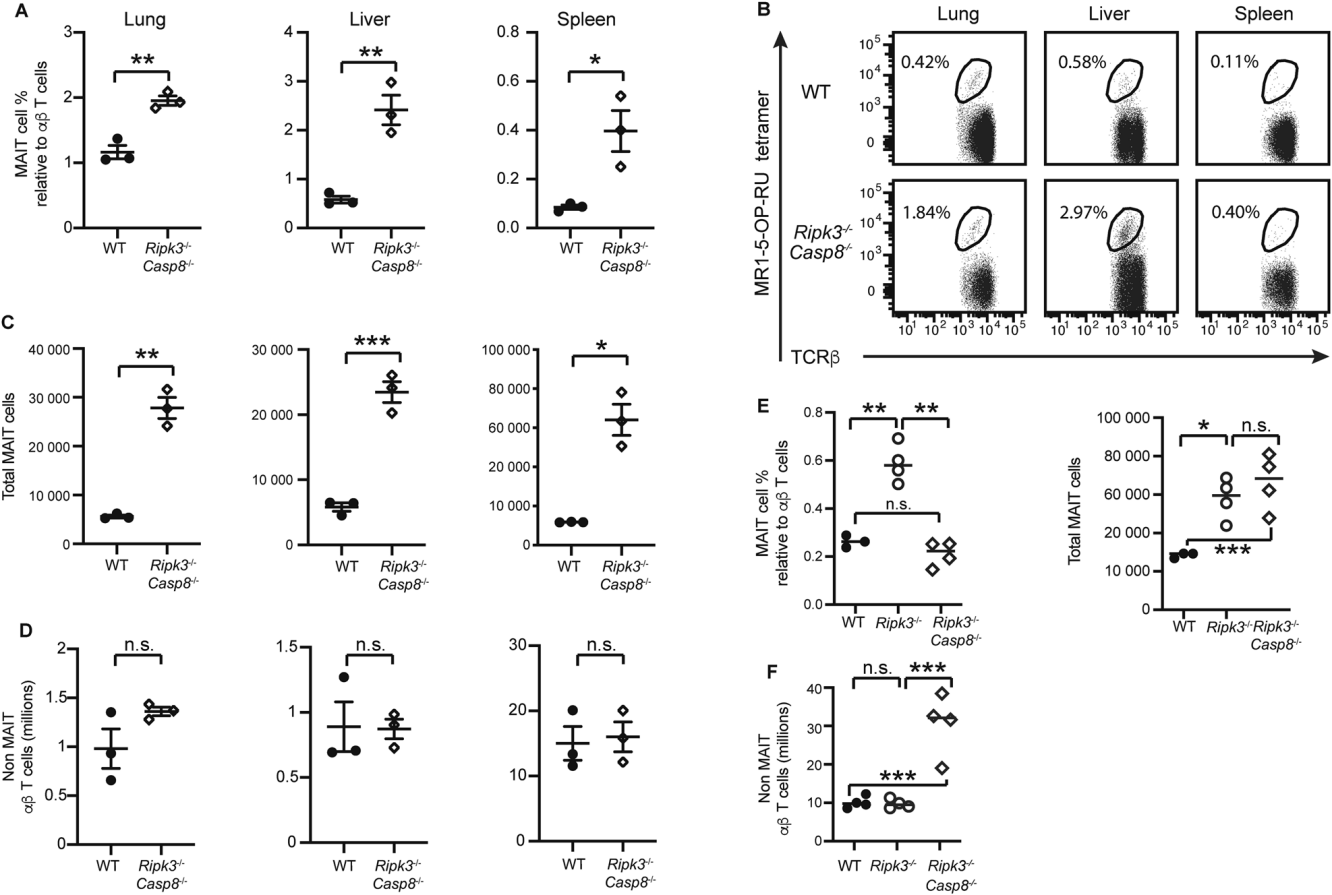
*Ripk3*^−/−^*Caspase8*^−/−^ mice and *Ripk3*^−/−^ mice have comparable increases in MAIT cells. **A–D** 6-8 week old C57BL/6 (WT) (*n* = 3) and *Ripk3*^−/−^*Caspase8*^−/−^ mice (*n* = 3) were analysed for T cell populations in the liver, lungs and spleen. **A** Relative abundance of MAIT cells, and **B** flow plots showing TCRβ^+^ lymphocytes with MAIT cells gated. MAIT cell abundance is expressed as a percentage relative to TCRβ^+^ lymphocytes. **C** Absolute number of MAIT cells and **D** absolute number of non-MAIT TCRβ^+^ lymphocytes. Statistical significance is indicated by ns (*p* > 0.05); * (*p* ≤ 0.05); ** (*p* ≤ 0.01); or *** (*p* ≤ 0.001) as determined by an unpaired two-tailed *t*-test with Welch’s correction for unequal variance. **E, F** Increase in MAIT cells and non-MAIT TCRβ^+^ cells in older *Ripk3*^−/−^*Caspase8*^−/−^ mice. Older (>16 weeks old) C57BL/6 (WT) (*n* = 3), *Ripk3*^−/−^ (*n* = 4) and *Ripk3*^−/−^*Caspase8*^−/−^ mice (*n* = 4) were analysed for MAIT and non-MAIT TCRβ^+^ cells in the spleen. (**E**) Graphs show the percentage and absolute number of MAIT cells and (**F**) the absolute number of non-MAIT TCRβ^+^ T cells. Statistical significance is indicated by ns (*p* > 0.05); * (*p* ≤ 0.05); ** (*p* ≤ 0.01); or *** *(p* ≤ 0.001) as determined by one-way Brown-Forsythe and Welch ANOVA, followed up with an unpaired two-tailed t-test with Welch’s correction for unequal variance.

Young *Ripk3*^*−/−*^*Casp8*^*−/−*^ mice exhibited an increase in the proportion and number of MAIT cells in the lungs, liver and spleen when compared to WT mice (Fig. 2A-D), which was equivalent to that observed in *Ripk3*^−/−^ mice (Fig. 1C, D). Importantly, *Ripk3*^*−/−*^*Casp8*^*−/−*^ and WT mice displayed comparable numbers of non-MAIT αβ T cells (Fig. 2D), indicating that the *Ripk3*^*−/−*^*Casp8*^*−/−*^ mice had not yet developed the autoimmune lymphoproliferative syndrome-like disease and associated accumulation of CD3^+^B220^+^CD4^−^CD8^−^ T cells that occurs in these mice upon aging [32**–**34]. Of note, in aged *Ripk3*^−/−^*Casp8*^−/−^ mice (>16 weeks), the accumulation of these aberrant non-MAIT TCRβ^+^CD4^−^CD8^−^ T cells rendered the percentage of spleen MAIT cells below that of *Ripk3*^−/−^ mice (Fig. 2E). Nevertheless, the absolute number of MAIT cells were increased similarly in both *Ripk3*^−/−^ mice and *Ripk3*^−/−^*Casp8*^−/−^ mice (Fig. 2E). Consistent with previous reports for conventional T cells [33], and distinct from *Ripk3*^−/−^ mice, non-MAIT αβ T cells expanded only in older *Ripk3*^−/−^*Casp8*^−/−^ mice (Fig. 2F).

**Fig. 3:**
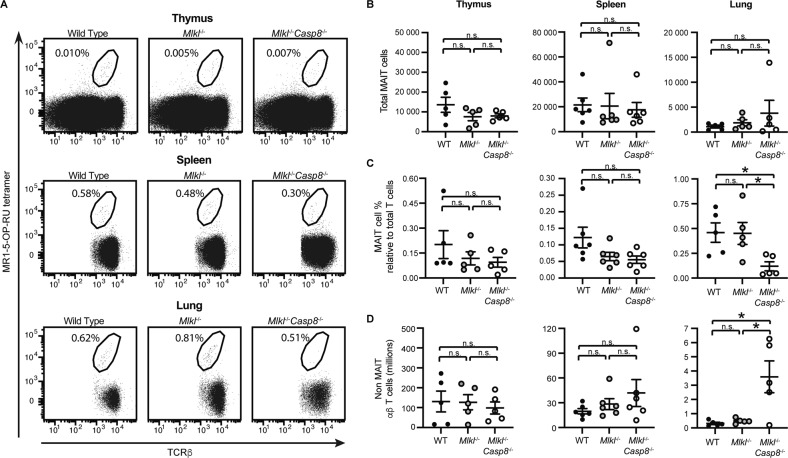
Neither the deficiency of the necroptotic effector MLKL, nor combined deficiency of both MLKL and the apoptotic effector Caspase-8, affect MAIT cell abundance. **A** Flow plots showing MAIT cell abundance in the thymus, spleen and lungs of 6-8 week old WT and *Mlkl*^−/−^ and *Mlkl*^−/−^*Casp8*^−/−^ mice. MAIT cell abundance is expressed as a percentage relative to TCRβ^+^ lymphocytes. **B** Absolute MAIT cell numbers, **C** relative MAIT cell abundance showing MAIT cell % relative to total thymocytes for the thymus, and MAIT cell % relative to αβ T cells for all other organs (as in **A**), and **D** absolute non-MAIT TCRβ^+^ lymphocytes in each of the indicated organs from WT (black circles), *Ripk3*^−/−^ (grey circles) and *Ripk3*^−/−^*Casp8*^−/−^ (hollow circles) mice. **B-D** Bars show the mean ± SEM (*n* = 6 mice), pooled from four independent experiments. Statistical significance is indicated by ns (*p* > 0.05); * (*p* ≤ 0.05); ** (*p* ≤ 0.01); or *** (*p* ≤ 0.001) as determined by one-way Brown-Forsythe and Welch ANOVA, followed up with an unpaired two-tailed t-test with Welch’s correction for unequal variance.

The observation that *Ripk3*^−/−^*Casp8*^−/−^ mice phenocopied RIPK3-deficient mice suggests either that RIPK3 signals contribute to MAIT cell thymic output and egress, independent of caspase-8; or that RIPK3 dominantly signals via caspase-8 to control MAIT cell abundance. Therefore, we next examined MAIT cell accumulation in mice lacking MLKL alone or MLKL and caspase-8 (*Mlkl*^−/−^ and *Mlkl*^−/−^*Casp8*^−/−^ mice, respectively) (Fig. S4A, B), which would allow investigation of whether caspase-8 or noncanonical RIPK3 signalling (i.e., independent of caspase-8 and MLKL) was regulating MAIT cell thymic output and egress. Strikingly, and unexpectedly, MAIT cell accumulation in organs of *Mlkl*^−/−^*Casp8*^−/−^ mice (Fig. 3A-C) did not recapitulate the accumulation of MAIT cells observed in *Ripk3*^−/−^ mice (Fig. 1B-D). Indeed, there was no significant difference in the total MAIT cell numbers in the thymus, spleen or lungs of *Mlkl*^−/−^ and *Mlkl*^−/−^*Casp8*^−/−^ mice when compared to age and sex-matched wildtype controls (Fig. 3B). In fact, in *Mlkl*^−/−^ and *Mlkl*^−/−^*Casp8*^−/−^ mice there was a trend towards a *decrease* in the abundance of MAIT cells relative to conventional T cells, which was statistically significant in the lungs (Fig. 3C); a likely effect of the accumulation of conventional T cells in these mice due to defective apoptotic and necroptotic signalling (Fig. 3D). Cumulatively, these results suggest that RIPK3 signalling is required for the maintenance of MAIT cell numbers during development and at steady state, in a manner independent of both caspase-8 and MLKL.

### RIPK3 but not MLKL regulates MAIT cell abundance during infection

**Fig. 4:**
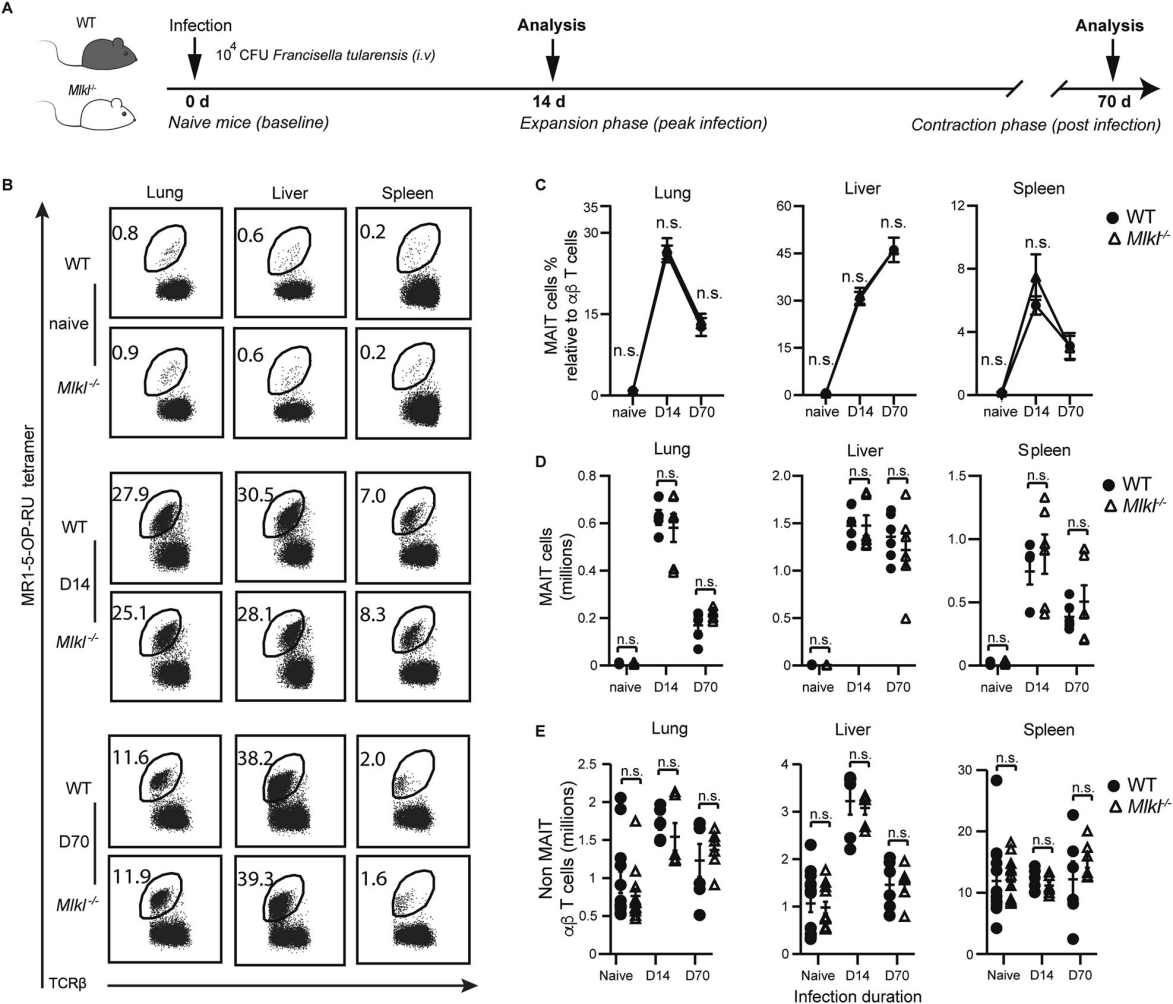
*Mlkl*^−/−^ mice have no increase in MAIT cell abundance during *F. tularensis* infection. **A** C57BL/6 (WT) and *Mlkl*^−/−^ mice were either uninfected or infected with 10^4^ CFU of *F. tularensis* LVS (i.v.) for 14 or 70 days. **B** Flow plots showing TCRβ^+^ lymphocytes in lungs, liver, and spleen from WT or *Mlkl*^−/−^, as indicated, with MAIT cells gated in naïve mice and at the indicated time points postinfection. MAIT cell abundance is expressed as a percentage of TCRβ^+^ lymphocytes. **C** Relative MAIT cell abundance (as in **B**) in infected and naïve WT and *Mlkl*^−/−^ mice at the indicated time points and in indicated tissues. **D** Absolute numbers of MAIT cells and **E** non-MAIT TCRβ^+^ lymphocytes in naïve and infected mice at the indicated time points and in the indicated tissues. **C-E** Error bars show the mean ± SEM of data pooled from 2 experiments (naïve mice *n* = 8; d14 infected *n* = 6; d70 infected *n* = 6). Statistical significance is indicated by ns (*p* > 0.05); * (*p* ≤ 0.05); ** (*p* ≤ 0.01); or *** (*p* ≤ 0.001) as determined by an unpaired two-tailed *t*-test with Welch’s correction for unequal variance.

Considering MLKL deficiency did not affect the accumulation of MAIT cells in the steady-state, we next considered a role for MLKL in the regulation of MAIT cell expansion and contraction during infection. Using a model of infection with *F. tularensis* live vaccine strain (LVS) [12], we examined MAIT cell expansion at 14 days post infection (dpi) and contraction following infection resolution at 70 dpi in WT and *Mlkl*^−/−^ mice (Fig. 4A). Both the absolute number and proportion of MAIT cells in the lungs and spleen of WT mice were elevated at 14 dpi (~30-fold from baseline) (Fig. 4B-D) and subsequently contracted by 70 dpi (Fig. 4B-D). In the case of the liver, MAIT cell abundance continued to increase and absolute numbers remained elevated at 70 dpi (Fig. 4B-D), despite a contraction of conventional αβ T cells in all organs (Fig. 4E). Critically, there was no significant difference in either the absolute number or relative abundance of MAIT cells between WT and *Mlkl*^−/−^ mice at baseline, 14 or 70 dpi (Fig. 4C, D), suggesting that necroptotic MLKL activity is not required for homeostatic maintenance, expansion or contraction of MAIT cells in *F. tularensis* infection.

**Fig. 5:**
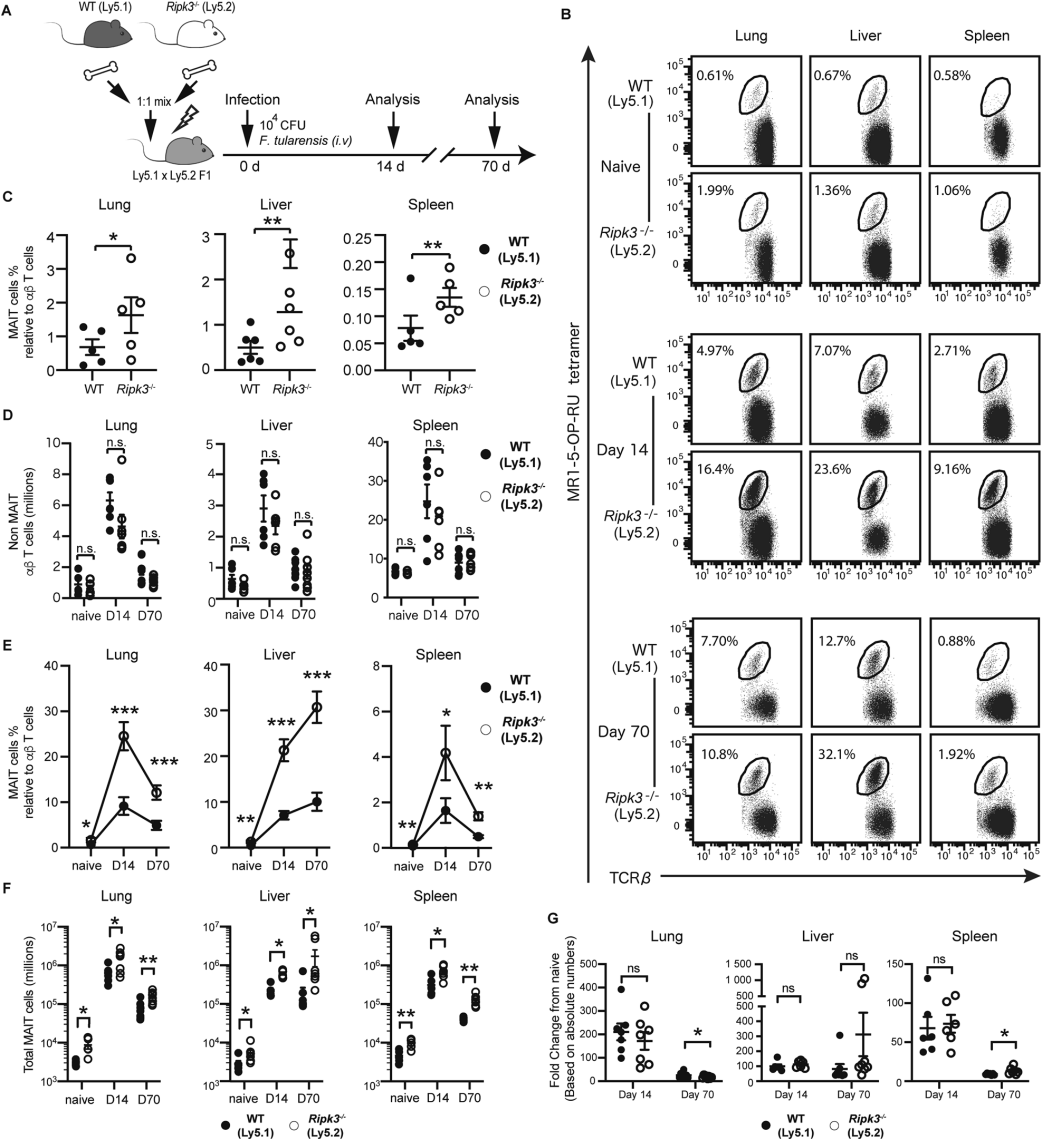
*Ripk3*^−/−^ MAIT cells are specifically expanded in mixed chimera mice at baseline and through infection. WT:*Ripk3*^−/−^ mixed bone marrow chimeric mice were generated by co-transfer of WT (Ly5.1) and *Ripk3*^−/−^ (Ly5.2) bone marrow, and then either uninfected or infected with 10^2^ CFU of *F. tularensis* LVS (i.t.) for 14 or 70 days, as indicated. **A** Schematic showing the experimental design. **B** Flow plots showing TCRβ^+^ lymphocytes with WT (Ly5.1) and *Ripk3*^−/−^ (Ly5.2) MAIT cells gated in naïve mice and at the indicated time points postinfection. MAIT cell abundance is expressed as a percentage relative to non-MAIT TCRβ^+^ lymphocytes. **C** Relative MAIT cell abundance (as in **B**) in uninfected mice showing WT (Ly5.1) and *Ripk3*^−/−^ (Ly5.2) from the same recipient mice. **D** Absolute numbers of MAIT cells and non-MAIT TCRβ^+^ T cells in naïve and infected mice. **E** Relative abundance of WT and *Ripk3*^−/−^ MAIT cells within chimeric mice, expressed as the percentage of TCRβ^+^ lymphocytes at indicated time points postinfection. **F** Absolute numbers of WT and RIPK3^−/−^ MAIT cells within chimeric mice, at indicated time points postinfection. **G** WT and *Ripk3*^−/−^ MAIT cells within chimeric mice expressed as fold change at day 14 and day 70 postinfection relative to uninfected mice. **C-G** Symbols show the mean ± SEM of data pooled from 2 independent experiments (naïve *n* = 5; d14 *n* = 7; and d70 *n* = 8). Statistical significance is indicated by ns (*p* > 0.05); * (*p* ≤ 0.05); ** (*p* ≤ 0.01); or *** (*p* ≤ 0.001) as determined by a paired two-tailed *t-*test.

As RIPK3 appeared to control MAIT cell thymic output and peripheral organ accumulation, we next queried whether cell intrinsic RIPK3 signalling versus extrinsic “indirect” factors (e.g. cytokine production) controlled MAIT cell abundance, and, importantly, whether RIPK3 also restrained MAIT cell expansion during infection. To answer these questions, we generated mixed bone marrow chimeras with a 1:1 mix of WT (Ly5.1) and *Ripk3*^−/−^ (Ly5.2) donor cells adoptively transferred into irradiated WT hosts, which would allow us to track whether any difference in MAIT cell responses were cell intrinsic, basally or during infection (Fig. 5A). Critically, we found that *Ripk3*^−/−^ (Ly5.2^+^) MAIT cells in chimeric mice significantly outnumbered the WT (Ly5.1^+^) by approximately 2-fold in the steady-state in all organs examined (Fig. 5B, C, F); a finding consistent with our global knockout analysis (Fig. 1B, C) and in line with the notion that RIPK3 regulates MAIT cell abundance in a cell-intrinsic manner. In contrast, there were no significant differences in the absolute numbers of *Ripk3*^−/−^ (Ly5.2^+^) and WT (Ly5.1^+^) non-MAIT αβ T cells in chimeric mice (Fig. 5D). Importantly, whilst the number of non-MAIT αβ T cells in these chimeras were comparable between WT and *Ripk3*^−/−^ cells throughout *F. tularensis* infection (Fig. 5D), the number and abundance of *Ripk3*^−/−^ MAIT cells relative to αβ T cells was significantly elevated at day 14 and day 70 post infection when compared to WT MAIT cells (Fig. 5E, F). However, when we accounted for the increase in MAIT cell abundance at baseline by expressing MAIT cell absolute counts for each genotype at day 14 and 70 post infection as a fold-change against the mean baseline count, we found no significant difference between WT and *Ripk3*^−/−^ mice in MAIT cell expansion or contraction (Fig. 5G), apart from in the lungs and spleen at day 70 where *Ripk3*^−/−^ MAIT cells exhibited a marginal decrease (in the lungs) and increase (in the spleen) compared to WT (Fig. 5G).

It remained important to understand the possible contribution of RIPK3 to non-death activities, such as inducing cytokine production, as described for other cell types [17, 19, 35–37]. As we have previously shown that *F. tularensis* polarises the responding MAIT cell population towards a MAIT-1 phenotype that confers protection from local and systemic infection [12], we next analysed IFN-γ production by WT and *Ripk3*^−/−^ MAIT cells and non-MAIT αβ T cells during infection in chimeric mice (Fig. S5). As expected, we observed a rapid expansion of IFN-γ producing MAIT and non-MAIT αβ T cells during the course of *F. tularensis* infection; particularly in the liver and lungs (Fig. S5A, B). The proportion of WT and RIPK3 deficient MAIT cells expressing IFN-γ remained comparable in chimeric animals (Fig. S5A, B).

Lastly, to ascertain if *Ripk3*^−/−^ MAIT cells were capable of conferring protection against *F. tularensis*, we expanded and subsequently transferred either WT or *Ripk3*^−/−^ MAIT cells, or no cells, into *Rag2*^−/−^*γc*^−/−^ mice (Fig. S6, A, B), as previously described [12]. This model allowed us to assess the impact of MAIT cell-specific RIPK3 deficiency, and to compare MAIT cell functionality in the absence of other immune cell populations that control bacterial infection, as *Rag2*^−/−^*γc*^−/−^ lack T, B and NK cells. Importantly, the transfer of *Ripk3*^−/−^ MAIT cells mirrored the expected WT MAIT cell transfer phenotype [12], as transferred *Ripk3*^−/−^ or WT MAIT cells equally conferred protection from weight loss (Fig. S6C) and significantly increased survival time post infection compared to the no transfer group (Fig. S6D). Critically, there was no significant difference in the survival time between the *Ripk3*^−/−^ MAIT and WT MAIT recipient groups, or in the bacterial load in the liver or spleen of mice transferred with *Ripk3*^−/−^ or WT MAIT cells that survived to day 19 post infection (Fig. S6E). Similarly, there was no significant difference in the absolute number, proportion or tissue density of MAIT cells, between the WT and *Ripk3*^−/−^ MAIT cell recipients surviving at endpoint (Fig. S6F), indicating equivalent expansion of MAIT cells post transfer and infection. Collectively, these results suggest that RIPK3 deficiency does not impact MAIT cell expansion, contraction, or MAIT-1 polarisation (at least in terms of their effector function through IFN-γ secretion) during infection, nor does loss of RIPK3 perturb MAIT cell capacity to control bacterial infection.

## Discussion

RIPK3 an be important for the regulation of extrinsic caspase-dependent and -independent cell death. However, loss of RIPK3 in mice does not lead to an abnormality in frequency of conventional T cells, B cells, natural killer cells, NKT cells or macrophages [29, 38]. *Ripk3*^−/−^ mice also appear healthy, are fertile, and live well into adulthood [29]. Here we reveal that under physiological conditions, loss of RIPK3 alone causes a consistent and significant increase in MAIT cells across the primary and secondary lymphoid organs, as well as peripheral tissues. This increase was specific to MAIT cells, with no changes in conventional αβ T cells, NKT cells or ILCs in *Ripk3*^−/−^ mice. RIPK3-mediated effects on MAIT cells were also cell-intrinsic as shown by the recapitulation of results in mixed bone marrow chimeras reconstituted with both WT and *Ripk3*^−/−^ cells.

After normalising for the increased accumulation of MAIT cells in the thymus, relative MAIT cell numbers in the secondary lymphoid and periphery were not significantly different between WT and *Ripk3*^−/−^ mice. Moreover, despite higher absolute numbers of MAIT cells in *Ripk3*^−/−^ mice than WT mice following infection of mice with *F. tularensis*, our fold change analysis suggested that the relative expansion and contraction of MAIT cells is unperturbed in the absence of RIPK3. These findings suggest that the function of RIPK3 in restraining MAIT cell accumulation controls thymic output and does not impact either homeostatic maintenance of MAIT cells or their expansion and contraction during infection. Further work is thus needed to understand how RIPK3 may regulate MAIT cell development in the thymus.

Consistent with their ‘*effector memory*’ like phenotype (CD44^hi^ CD62L^lo^), both MAIT and non-MAIT effector memory αβ T cells expressed an abundance of the necroptotic effector MLKL, unlike naïve non-MAIT αβ T cells. Despite this observation, the increase in MAIT cell accumulation in *Ripk3*^−/−^ mice was not phenocopied in *Mlkl*^−/−^ or *Mlkl*^−/−^*Casp8*^−/−^ mice, suggesting a novel mechanism for RIPK3 in restraining MAIT cell accumulation, independent of canonical apoptotic caspase-8 and MLKL-driven necroptotic cell death pathways. These findings resonate with the recent work of Teh and colleagues, in which both human and mouse T_regs_ were shown to exhibit increased expression of MLKL and RIPK3 relative to their conventional counterparts, but nonetheless remained resistant to necroptosis following either TCR engagement or stimulation with canonical necroptotic stimuli [25]. Indeed, it was shown that T_regs_ were only susceptible to necroptosis following pre-priming with IL-12, IL-18 or IFN-β [25], which was postulated to regulate post-translational activation of the pathway. As each of these cytokines have previously been implicated in the immune response to *F. tularensis*, it remains possible that MAIT cells are susceptible to necroptosis under similar conditions, but that in our in vivo model of *F. tularensis* LVS infection we did not induce sufficient inflammation to activate this pathway.

Considering the abundant expression of both RIPK3 and MLKL, it remains possible that RIPK3 is capable of engaging MLKL to limit MAIT cell numbers. It is enticing to speculate that RIPK3 exerts MLKL-independent actions to restrict thymic MAIT cell production and egress. One such alternate pathway is the Calcium/Calmodulin-dependent kinase II (CaMKII) cell death pathway, which can be activated by RIPK3 activity [39**–**41]. The CamKII pathway is well-reviewed for its pleiotropic role in regulating both noncanonical apoptotic and necroptotic functions in cardiac tissue [41, 42], while also regulating several non-death functions in T cells [43, 44] and promoting survival of double positive (DP) thymocytes during development [44].

While our results implicate RIPK3 in restraining MAIT cell accumulation during thymic development, it is as yet unclear why MAIT cells, relative to conventional T cells, preferentially depend on RIPK3. The simplest answer would be that the relative expression of RIPK3, or a key novel downstream target, dictates the signalling outcome. In cancer cell lines that ubiquitously express RIPK1, the availability of RIPK3 correlates with their sensitivity to necroptosis induction [45]. Alternatively, it may be that the scaffolding function of RIPK3, and not its kinase activity, is required to restrain MAIT cell accumulation. It has been previously shown that the scaffolding function, and not the kinase activity, of the closely related RIPK1 is required for the survival of conventional T cells in the secondary lymphoid organs, while the immature T cell compartment in the thymus remains unaffected upon RIPK1 or RIPK1 kinase activity loss [46]. In contrast, our findings suggest a divergent role for RIPK3 in the regulation of MAIT cell survival during development, in that the increased abundance appears to originate in the thymus. Considering that the Vα19 gene is located at the distal end of the TCR locus and one of the last to re-arrange [47], it is also possible that RIPK3 restrains the lifespan of DP thymocyte precursors and thus allows for the greater generation of MAIT cells. Indeed, the opposite is true of RORγt-deficient mice which exhibit a decreased DP thymocyte lifespan and lack of Vα19 transcripts [48]. Therefore, further work is required to understand the mechanisms by which RIPK3 restrains MAIT cell accumulation during thymic development, and what dictates the specificity to MAIT cells and not other unconventional or conventional T cell subsets.

Cumulatively, we have shown that RIPK3 plays a critical role in the regulation of MAIT cell abundance under physiological conditions at steady state. Our findings point towards a specific role for RIPK3 in the regulation of MAIT cell accumulation during thymic development, which is independent of both MLKL-mediated necroptosis and caspase-8-mediated apoptosis and thus warrants further investigation. Considering MAIT cells have been increasingly implicated in protective immunity [8, 10, 11], as well as in driving immunopathology [13, 14], our insights into their developmental regulation might open up new avenues for immune intervention.

## Materials and methods

### Mice

C57BL/6 (B6, WT), Ly5.1, Ly5.1/Ly5.2 F1, *Ripk3*^−/−^ [29], *Mlkl*^−/−^ [20], *Caspase-8*^−/−^/*Mlkl*^−/−^*, Caspase-8*^−/−^*/Ripk3*^−/−^ [33] and *Rag2*^−/−^γc^−/−^ mice were housed under specific pathogen-free conditions at the Walter and Eliza Hall Institute of Medical Research (WEHI, Melbourne, Australia) or the Biological Research Facility of the Doherty Institute (Melbourne, Australia). Mixed chimera mice were generated by irradiating Ly5.1/Ly5.2 F1 mice with 2 doses of 550 rad and reconstituting recipients with 2 × 10^6^ BM cells (1:1 mix from Ly5.1 WT and Ly5.2 *Ripk3*^−/−^ mice). For experiments involving multiple treatment groups, cages of mice were randomly allocated to groups. The investigator was blinded to the group allocation for the assessment of survival (based on humane endpoints) following infection but was not blinded for experiments involving flow cytometric analysis of cells from mice. No samples were excluded from analysis.

### Bacterial culture and inoculation

*Francisella tularensis* live vaccine strain (LVS) was grown in brain heart infusion (BHI) broth and cultured for 16-18 h at 37 °C with continuous shaking at 180 rpm. The concentration of bacteria was estimated by determining the optical density (OD) values at 600 nm where 1 OD_600_ ≈ 2.4×10^9^ CFU/ml. For inoculation of mice, the bacteria were washed and diluted to the necessary concentration with PBS. Mice were infected with 1×10^4^ - 2×10^4^ CFU in 200 μl intravenously (i.v.) or 50 CFU in 50 μl intratracheally (i.t.). *Rag2*^−/−^γc^−/−^ are severely immune compromised, so were inoculated with 40 CFU *F. tularensis* LVS intravenously (i.v.). The inoculum dose was verified by plating remaining inoculum on cysteine heart agar with 10 μg/ml ampicillin, 7.5 μg/ml colistin and 4 μg/ml trimethoprim and counting colony forming units (CFU) after incubation at 37 °C for 4 days.

### Cell isolation

Isolation of single cells from mouse liver, lung, spleen and intestine was performed essentially as described [49]. *Liver*. Prior to dissection, livers were perfused postmortem with 10 mL cold RPMI injected via the portal vein. To prepare single-cell suspensions, liver tissue was pushed through 70 μm cell strainers. Red blood cells were lysed using tris ammonium chloride hypotonic lysis buffer (TAC; 0.14 M NH_4_Cl, 0.017 mM Tris, pH 7.2) and the cells were re-suspended in 12 mL 33.75% Percoll, underlaid with 12 mL 70% percoll and centrifuged for 20 min at 900 *g* at room temperature with a low brake. The cell pellets were then washed, re-suspended in FACS buffer [i.e., PBS containing 2% fetal calf serum (FCS; Bovogen Biologicals, Keilor East, Australia)] and filtered through 100 μm cell strainers. *Lungs*. Lungs were perfused prior to dissection by injecting 10 mL cold RPMI via the right ventricle of the heart. The lungs were chopped into fine pieces with surgical scalpel blades, placed into 1 mL RPMI containing 3 mg/mL collagenase III (Worthington, Lakewood, NJ USA), 5 μg/mL DNase I and 2% (v/v) FCS and incubated for 90 min at 37 °C with continuous shaking at 180 rpm, before being filtered through 70 μm strainers. *Spleen*. Splenocytes were isolated by gently homogenising spleens through a 70 μm cell strainer, red blood cells were lysed with TAC, and cells re-suspended in FACS buffer and filtered through 100 μm strainers. *Intestine*. To isolate intestinal cells, adipose tissue and Peyer’s Patch were removed from the small intestine, the mucosal surface was cleaned and tissues were cut into ~2 cm sections. Small fragments were then dissociated in HANKS Ca^2+^Mg^2+^ Free Media supplemented with 5 mM EDTA and 2% FCS, for 40 min at 37 °C with gentle agitation as previously described [50, 51]. The dissociated epithelial layer was then discarded and remaining gut fragments were incubated in 1 mg/mL Collagenase IV (Worthington), 200 μg/mL DNase I (Roche) and 4 U/mL Dispase (Sigma, St Louis, MO, USA) for 45 min at 37 °C with gentle agitation. Lamina propria mononuclear immune cells were isolated by centrifugation on a 40%-80% Percoll gradient. Lymphocytes were recovered from the interface and washed in cold FACS buffer prior to staining.

### MAIT cell isolation and adoptive transfer

To increase MAIT cell numbers in mice prior to isolation and transfer, donor WT and *Ripk3*^−/−^ mice were immunised with 5-(2-oxopropylideneamino)-6-D-ribitylaminouracil (5-OP-RU, 2 nmol [52, 53],) and CpG (1 nmol, T*C*G*T*C*G*T*T*T*T*G*T*C*G*T*T*T*T*G*T*C*G*T*TT*CG*T*CG*A*CG*A*T*CG*G*C*G*CG*C*G*C*C*G; *phosphorothioate linkage, non-methylated cytosine-guanosine oligonucleotides, Integrated DNA Technologies, Singapore) over 7 days to boost MAIT cells systemically, as previously described [5, 12, 49]. MAIT cells were FACS sorted (gating shown in Fig S1) and 8 × 10^4^ MAIT cells transferred to recipient mice by i.v. injection in 200 μl PBS. Recipient mice were subsequently treated twice over three days with intraperitoneal injection of α-CD4 (GK1.5; 100 μg) and α-CD8 (53.6-7; 100 μg) to deplete any contaminating transferred conventional T cells. Two weeks post transfer, mice were infected with 40 CFU (i.v.) of *F. tularensis* and weight and survival (based on humane endpoints including weight loss and loss of body condition) monitored daily for 19 days.

### Mouse antibodies and flow cytometry

To stain for T cell subsets single cell suspensions were prepared from the lungs, liver, spleen and thymus, and stained with Fixable Viability Dye (FVD) eFluor780 on ice for 30 min (Invitrogen, Waltham, MA, USA; #65-0865-18; 1/1,000). Prior to surface staining, non-specific staining was blocked with blocking solution (2.4G2 hybridoma supernatant; in house) containing MR1-6-formyl pterin (6-FP) control tetramer at 1:100 (unlabelled, in house) [54] for 15 min at room temperature in the dark. MR1 tetramers were prepared as described previously [7]. Cells were subsequently stained for 30 mins at room temperature in the dark with a cocktail of antibodies, at indicated dilutions: anti-mouse CD45-BV711 (BioLegend, San Diego, CA, USA; #103147; Clone 30-F11, 1/400), anti-mouse CD45.1-FITC (BioLegend #110706; Clone A20, 1/200), anti-mouse CD45.2-FITC (BD, Franklin Lakes, NJ, USA; #553772; Clone 104, 1/400) anti-mouse CD45.2-APCeFluor780 (Invitrogen #47-0454-82; Clone 104, 1/200), anti-mouse CD19-PerCP-Cy5.5 (BD #551001; Clone 1D3, 1/200), anti-mouse CD19-AlexaFluor700 (BioLegend #115528; Clone 6D5, 1/200), anti-mouse TCRβ-FITC (BD #553171; Clone H57-957, 1/300), anti-mouse TCRβ-APC (BD #553174, Clone H57-597 1/200), mouse MR1-5-OP-RU tetramer (BV421/PE) (in house), α-galactosylceramide loaded CD1d tetramers (PE, provided courtesy of Dale Godfrey), anti-mouse CD4-APC (BioLegend #100412; Clone GK1.5, 1/200), anti-mouse CD4-BV786 (BD #563331; Clone GK1.5, 1/200), anti-mouse CD8α−PE (Invitrogen #12-0081-82; Clone 53-6.7, 1/220), anti-mouse CD8α−PE-Cy7 (BD #552877; Clone 53-6.7), anti-mouse CD44-BUV737 (BD 612799; Clone IM7, 1/200) anti-mouse CD44-FITC (BD #553133; Clone IM7 1/200), anti-mouse CD62L-BUV395 (BD #740218; Clone MEL-14, 1/200) and IFN-γ-PE-Cy7 (BD #557649; Clone XMG1.2, 1/200). Finally, cells were washed twice with FACS buffer (PBS supplemented with 2% FCS and 2 mM EDTA) and re-suspended in PBS supplemented with 2 mM EDTA and 2% FCS, or a fixative solution of PBS containing 2% w/v D-glucose and 1% paraformaldehyde. Sphero blank calibration beads (BD #556926) were added as per manufacturer’s instructions prior to analysis for the quantitation of cell numbers.

To stain for ILCs, single-cell suspensions of lung and intestine preparations were stained with the following antibodies: anti-mouse CD11b-BV510 (BD #562950; Clone M1/70, 1/800), anti-mouse CD49a-BV711 (BD #563863; Clone Ha31/8, 1/300), anti-mouse CD3ε−BUV395 (BD #563565; Clone 145-2C11, 1/200), anti-mouse CD19-BUV737 (BD #612781; Clone 1D3, 1/800), anti-mouse CD45-BV786 (BD #564225; Clone 30-F11, 1/200), anti-mouse NK1.1-BV650 (BD #564143; Clone PK136, 1/200), anti-mouse KLRG1-AF647 (eBioscience, Waltham, MA, USA; #51589382; Clone 2F1, 1/200), NKp46-PECy7 (eBioscience #25-3351-82; Clone 29A1.4, 1/100), TCRβ−APCeFluor780 (Invitrogen #47-5961-82; Clone H57-597, 1/200) together with the fixable viability dye eFluor^TM^ 780 (1/1000).

For intracellular cytokine staining (ICS) cells were stimulated with 20 ng/ml PMA and 1 μg/ml ionomycin for 3-4 h (37 °C, 5% CO_2_) in the presence of 10 µg/ml Golgi plug (BD). Surface stains were performed as described above, and intracellular cytokines subsequently stained with Cytofix/Cytoperm^TM^ Fixation/Permeabilisation Kit (BD) as per manufacturers’ instructions.

For transcription factor staining, cells were stained with surface markers and viability dye, as described above, then fixed and permeabilized using the FOXP3 eBioscience Kit (eBioscience #00-5523-00) for 30 min on ice. Cells were washed and then stained for 30-40 min with the following antibodies: anti-mouse GATA3-PE (eBioscience #12-9966-42; Clone TWAJ, 1/100), anti-mouse RORγt-BV421 (BD #562894; Clone Q31-378, 1/300) and anti-mouse- Eomes-PerCPeFluor-710 (Invitrogen #46-4785-82; Clone Dan11mag, 1/300).

Flow cytometric data for all staining protocols were acquired on a BD LSR Fortessa X20 (BD Bioscience) and analysed using Flowjo V10.8.1 software (BD). Fluorescence-activated cell sorting (FACS) to isolate T cell populations was performed on a FACS Aria III (BD) with assistance from staff at the University of Melbourne Cytometry Platform.

### Western blotting

Non-MAIT CD4 and CD8 T cells (TCRβ^+^) were sorted into naive and memory populations with antibodies to mouse CD4, CD8, CD44 and CD62L (Fig S1). MAIT and iNKT cells were gated based on the expression of TCRβ and reactivity with either MR1-5-OP-RU or CD1d-αGalCer tetramers, respectively (Fig S1). Bulk non-MAIT conventional αβ T cells were FACS sorted as live B220^−^TCRβ^+^ cells. Purified cell populations were lysed for 30 minutes on ice with radioimmunoassay precipitation assay (RIPA) buffer (150 mM sodium chloride; 1% [v/v] Triton X-100; 1% [w/v] sodium deoxycholate; 0.1% [w/v] SDS; in 10 mM Tri-HCl) supplemented with cOmplete protease and cOmplete phosphatase inhibitor tablets (Roche) as per manufacturer’s instructions. Lysates were diluted with 4× Novex NuPage LDS Sample Buffer (ThermoFisher Scientific; Waltham, MA, USA) supplemented with beta-mercaptoethanol (Sigma; final concentration 1.25% [v/v]), and examined by routine Western blotting as previously described [19]. Apoptotic and necroptotic machinery was probed from mouse cell lysates with the following antibodies: rabbit anti-mouse RIPK1 (Cell Signaling Technologies, Danvers, MA, USA; #3493 S; Clone D94C12, 1/1,000), rabbit anti-mouse RIPK3 (ProSci #2283; Polyclonal, 1/1000), rat anti-mouse MLKL (Abcam, Cambridge, UK; #ab243142; Clone 3H1, 1/1,000), or rat anti-mouse caspase-8 (Enzo Life Sciences, New York, NY, USA; #ALX-804-448-C100; Clone 3B10, 1/1,000); and detected with donkey anti-rabbit IgG Horse Radish Peroxidase (HRP) (Merck, Kenilworth, NJ, USA; #NA-934; 1/2,000) or goat anti-rat IgG HRP (SouthernBiotech, Birmingham, AL, USA; #4030-05; 1/2,000) as appropriate. For housekeeping, Hsp90 was probed with rabbit anti-mouse Hsp90 (Cell Signalling Technology #4874; 1/1,000) and detected with donkey anti-rabbit IgG HRP (1/2,000). β-actin was detected with HRP-conjugated rabbit anti-mouse β-actin (Cell Signaling Technology #4970; Clone 13E5, 1/10,000). Blots were developed with Imobilon Forte Western HRP substrate as per manufacturer’s instructions and imaged on a ChemiDoc Touch Gel Imaging System (BioRad, Hercules, CA, USA), and analysed Image Lab Software (BioRad; version 6.1.0).

### Statistical analysis

Sample sizes were estimated based on previous extensive experience in the laboratory within similar studies. All statistical tests were performed using Prism software (Version 9.3.1, GraphPad Software, LLC). Unless otherwise indicated all graphs show the mean and standard error of the mean (SEM). Comparisons between groups were made using either an unpaired two-tailed *t*-test with Welch’s correction for unequal variance, or a Brown-Forsythe and Welch ANOVA followed by an unpaired two-tailed *t*-test with Welch’s correction for unequal variance, as indicated. Survival data in MAIT cell adoptive transfer experiment were plotted using a Kaplan-Meier simple survival curve analysis, and significance determined using a log-rank (Mantel-Cox) test.

## Supplementary information

Supplementary Figures

Full length Western Blots

checklist

## Data Availability

All datasets generated and analysed during this study are included in this published article and its Supplementary Information files. Full length uncropped western blots are available in the supplemental material files. Additional data are available from the corresponding author on reasonable request.
