# Supplementary material for: RIPK3 controls MAIT cell accumulation during development but not during infection
Source: Cell Death Dis. 2023 Feb 11;14(2):111. doi: 10.1038/s41419-023-05619-0 (PMC9922319; doi:10.1038/s41419-023-05619-0)
Supplement: Supplementary file 1 — Supplementary Figures [file 41419_2023_5619_MOESM1_ESM.docx]

**
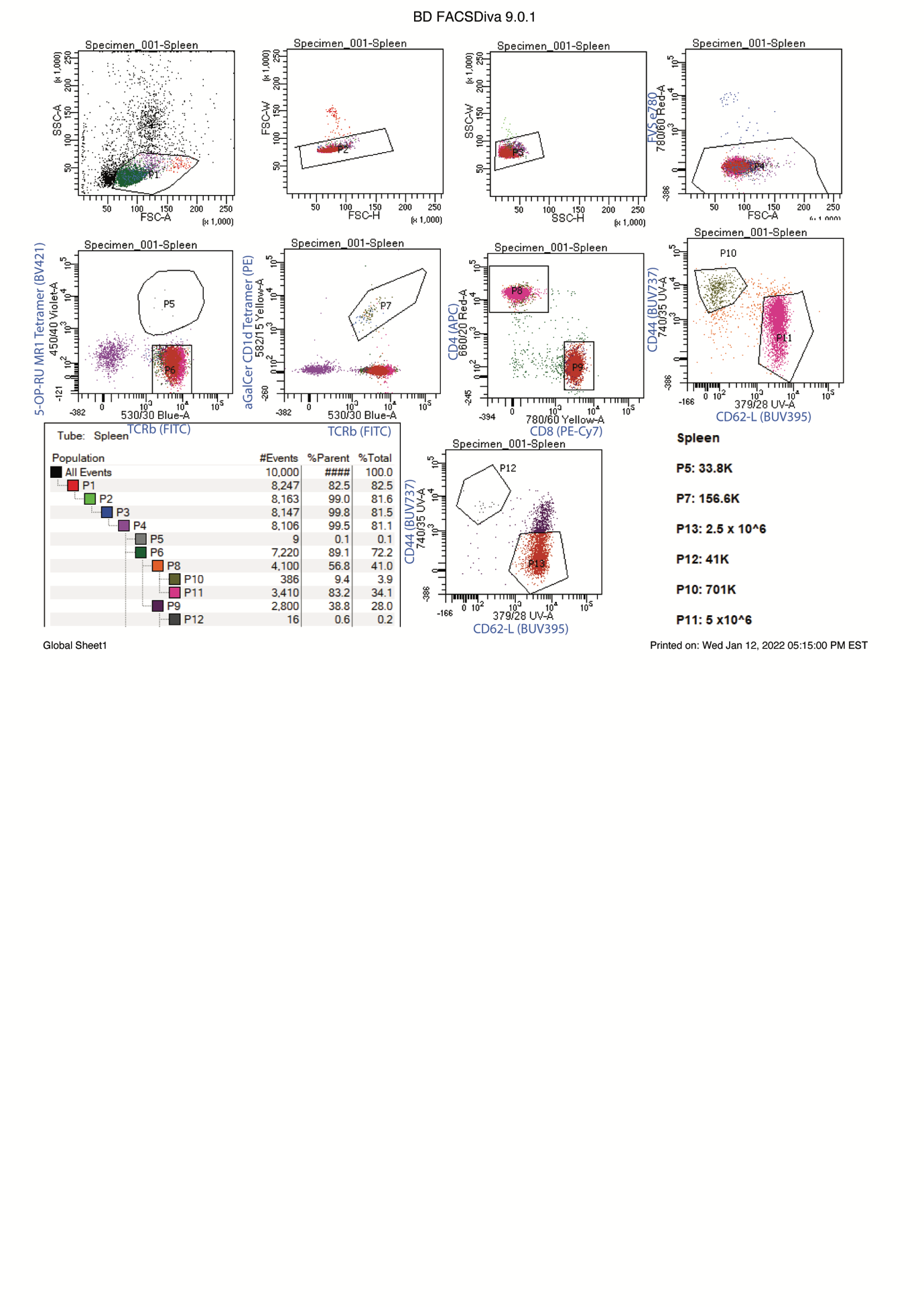
Figure S1. Gating strategy for FACS sorting of conventional T cell subsets and unconventional T cells**. **A** Dot plots showing sequential gating (left to right) of splenic MAIT cells (P5), iNKT cells (P7), conventional CD4^+^ naïve (P9) and memory (P10) T cells, conventional CD8^+^ naïve (P13) and memory (P12) T cells. Antibodies and fluorochromes are annotated in light blue, and relative abundance of each population is shown in the table.

**
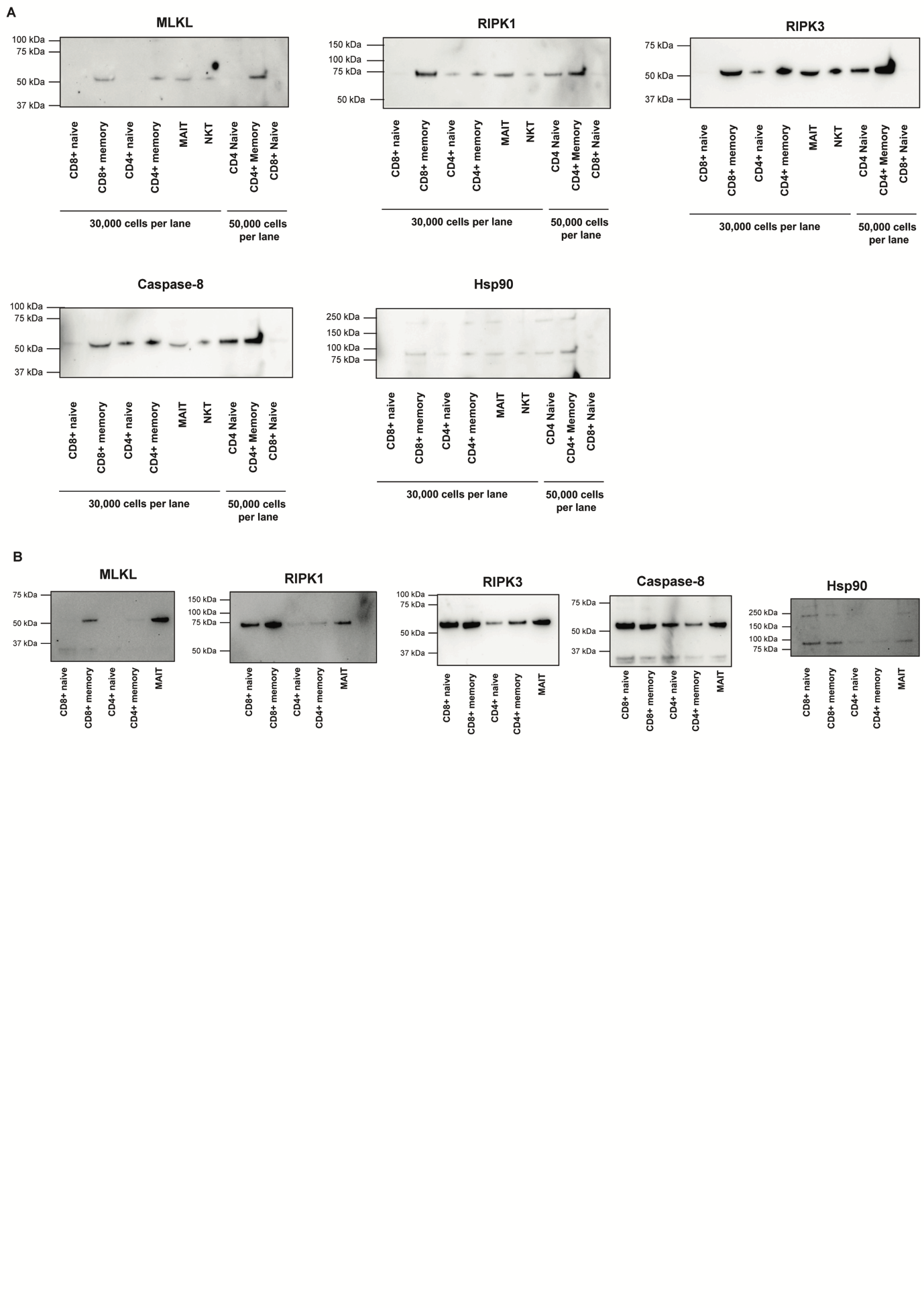
Figure S2. Expression profile of apoptotic and necroptotic machinery in conventional and non-conventional T cell populations. A**Western blots of MLKL, Caspase-8, RIPK1, RIPK3 and the Hsp90 house keeping control. Lanes loaded left to right with the with 5×10^4^ CD8^+^ naïve, CD8^+^ memory, CD4^+^ naïve and CD4^+^ memory conventional T cells, MAIT cells and iNKT cells; each FACS sorted from 12 pooled spleens. **B** Full Western blots of proteins as indicated in A, with lanes loaded (left to right) with 5×10^4^ CD8^+^ naïve, CD8^+^ memory, CD4^+^ naïve and CD4^+^ memory conventional T cells, and MAIT cells; each FACS sorted from spleens pooled from four mice four weeks after MAIT cell boosting with 5-OP-RU and CpG.

**
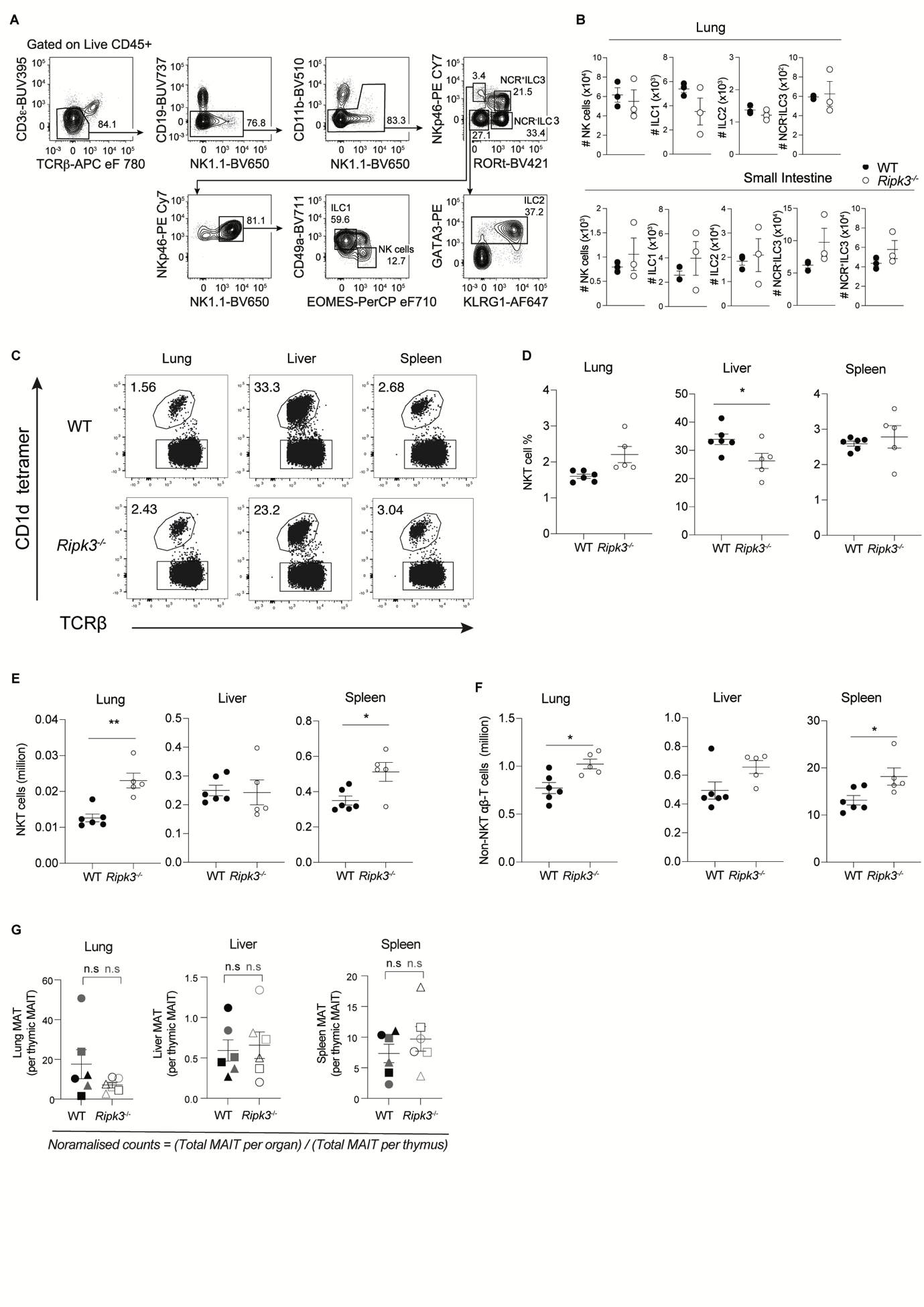
Figure S3 The effect of RIPK3 on MAIT cell accumulation is specific to MAIT cells**. **A** Representative contour plots showing the gating strategy used to identify ILC populations from the lamina propria of the small intestine. **B** Enumeration of NK cells, ILC1, ILC2, NCR^-^ILC3 and NCR^+^ILC3 (small intestine only) from the lung and small intestine of WT and *Ripk3^‑/‑^* mice. A similar gating strategy was used for the identification of lung ILCs. Scatter plots showing total number of NK cells and ILC subsets in the lung and small intestine. Data shows the mean ± SEM of WT and *Ripk3^-/-^* mice (n = 3). **C** Representative FACS plots, and scatter plots showing **D** the percentage and **E** the total number of NKT cells in the lung, liver and spleen of naïve WT and *Ripk3^-/-^* mice. Data shows the mean ± SEM of WT mice (n = 6) or *Ripk3^-/-^* mice (n = 5). **F** Total number of non-NKT αβ T cells in the lung, liver and spleen of naïve WT and *Ripk3^-/-^* mice. Data shows the mean and SEM (n = 6). **G** Thymic MAIT cell density (MAIT cells per mg of tissue) calculated from MAIT cell counts and thymic mass as presented in Fig. 1 C, F. Statistical significance is indicated by ns (*p* > 0.05); * (*p*≤ 0.05); ** (*p* ≤ 0.01); or *** (*p* ≤ 0.001) as determined by an unpaired two-tailed *t*-test with Welch’s correction for unequal variance.

**
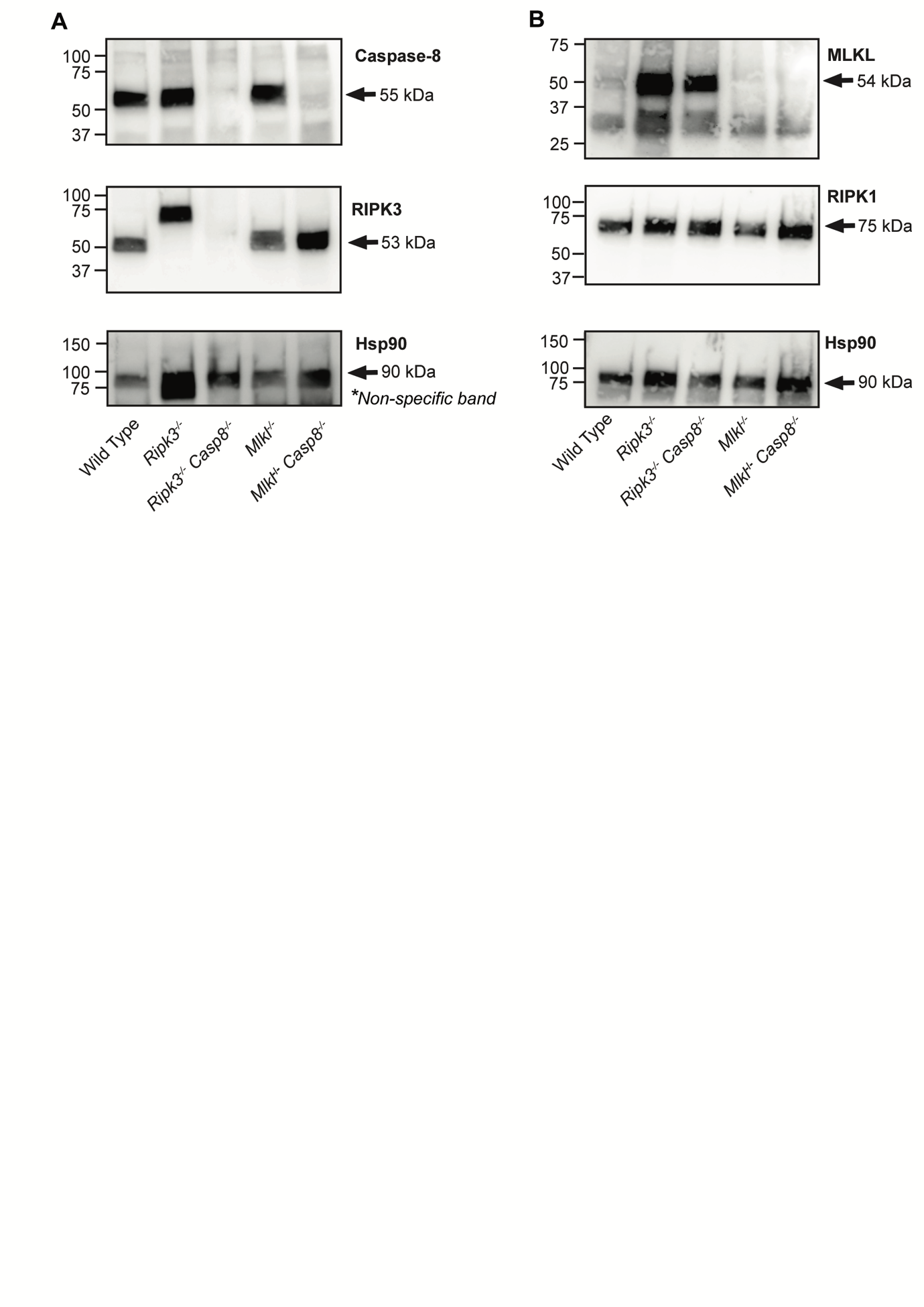
Figure S4 Western blots confirm no expression of Caspase-8, RIPK3 and MLKL in genetic knockout strains of mice. A** Western blot showing expression of caspase-8, RIPK3 and Hsp90 from 2 × 10^5^ total splenic T cells (B220^-^, TCRβ^+^) FACS sorted from wild-type, *Ripk3^-/-^, Ripk3^-/-^Casp8^-/-^, Mlkl^-/-^* and *Mlkl^-/-^Casp8^-/-^* mice. Membrane was probed first for caspase-8 and subsequently re-probed for RIPK3 then Hsp90. *Due to antibody species similarity, the re-probe of Hsp90 shows an unknown non-specific band at ~75 kDa which first appeared in the RIPK3 probe. **B** Western blot showing expression of MLKL, RIPK1 and Hsp90 from 2 × 10^5^ total splenic T cells (B220^-^, TCRβ^+^) FACS sorted from wild-type, *Ripk3^-/-^, Ripk3^‑/-^Casp8^-/-^, Mlkl^-/-^* and *Mlkl^-/-^Casp8^-/-^* mice. Membrane was probed first for MLKL and subsequently re-probed for RIPK1 then Hsp90.

**
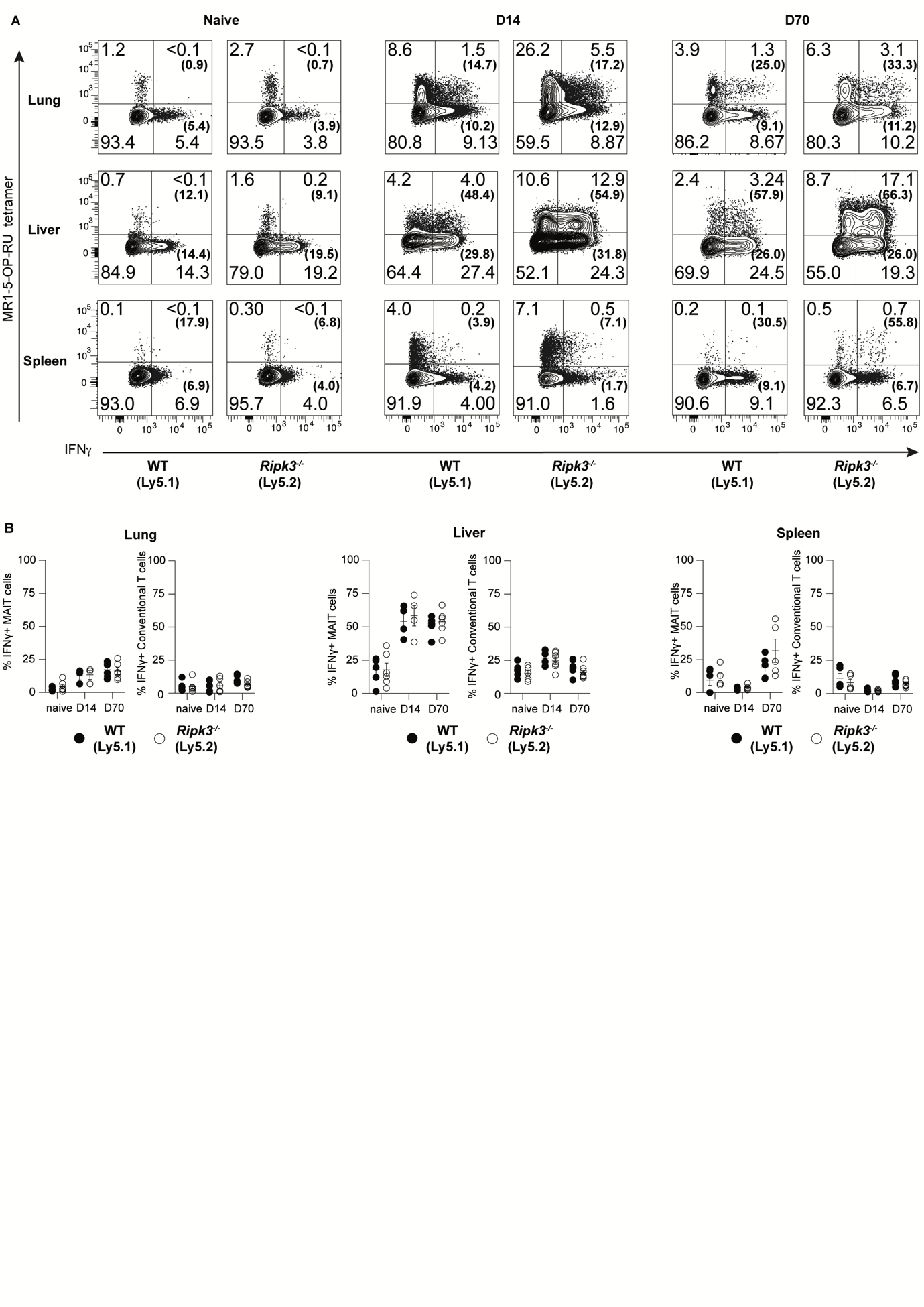
Figure S5 RIPK3 deficiency has no impact on IFN-γ production by MAIT cells**. **A, B** WT:*Ripk3^-/-^* mixed bone marrow chimeric mice either uninfected (naïve) or infected with 10^2^ CFU of *F. tularensis* LVS (i.t.) after 14 or 70 days. Cytokine production by T cells was determined after 4 h *in vitro* stimulation with PMA and ionomycin. **A** Flow cytometry plots show WT (Ly5.1) and *Ripk3^-/-^* (Ly5.2) TCRβ^+^ lymphocytes, derived from the same recipient mice, stained for IFN-γ vs MR1-5-OP-RU-tetramer. Numbers in the plots show percentages of cells in individual quadrants. Numbers in parentheses show the percentages of IFN-γ normalized to corresponding T cell subsets (MAIT or non-MAIT). **B** Graphs show the percentages of IFN-γ^+^ MAIT and non-MAIT T cells from four individual mice (n = 4).

**
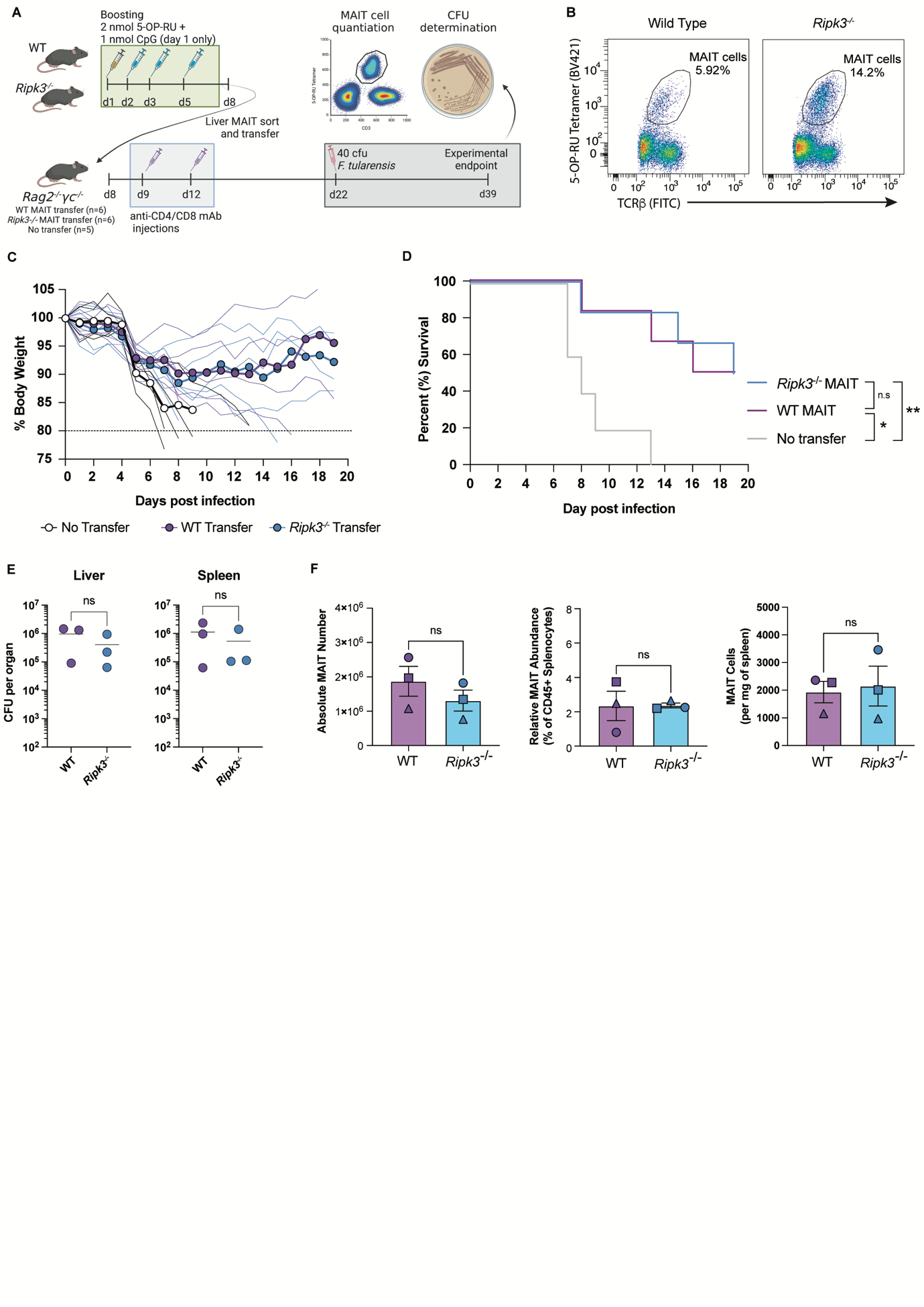
Figure S6 The transfer of *Ripk3^-/-^* or WT MAIT cells into *Rag2^-/-^γc^-/-^* mice is equally protective against *F. tularensis* infection. A** Schematic overview of experiment. Created with BioRender. **B** Abundance of MAIT cells in WT and *Ripk3^-/-^* livers FACS sorted for transfer to *Rag2^-/-^γc^-/-^* mice following boosting with CpG and 5-OP-RU as in A. **C** Weight curve showing average (thick lines) and individual (thin lines) percent (%) change in body weight of *Rag2^‑/‑^γc^‑/‑^* mice following *F. tularensis* infection, in the WT MAIT transfer group (purple line; n = 6), *Ripk3^-/-^* MAIT cell transfer group (blue line; n = 6) and no MAIT transfer group (grey line; n = 5). **D** Survival curve showing percent (%) survival of *Rag2^-/-^γc^-/-^* mice following *F. tularensis* infection, in the WT MAIT transfer group (purple line; n = 6), *Ripk3^-/-^* MAIT transfer group (blue line; n = 6) and no MAIT transfer group (grey line; n = 5). Statistical significance is indicated by ns (*p* > 0.05); * (*p*≤ 0.05); ** (*p* ≤ 0.01); or *** (*p* ≤ 0.001) as determined by a Kaplan-Meier simple survival curve analysis, comparing survival curves with a log-rank (Mantel-Cox) test. **E** Bacterial burden (CFU) in the liver and spleen of *Rag2^‑/‑^γc^-/-^* mice that received WT or *Ripk3*^‑/-^ MAIT cells surviving through day 19 post infection with *F. tularensis*. Statistical significance is indicated by ns (*p* > 0.05); * (*p*≤ 0.05); ** (*p* ≤ 0.01); or *** (*p* ≤ 0.001) as determined by an unpaired two-tailed *t*-test with Welch’s correction for unequal variance. **F** Splenic MAIT cells in *Rag2^-/-^γc^-/-^* recipient mice surviving at day 19 following *F. tularensis* infection. Data show absolute MAIT cell numbers (left), relative MAIT abundance (middle) and MAIT cell density from mice that received *Ripk3^-/-^* or WT MAIT cells (n = 3). Statistical significance is indicated by ns (*p* > 0.05); * (*p*≤ 0.05); ** (*p* ≤ 0.01); or *** (*p* ≤ 0.001) as determined by an unpaired two tailed *t*-test with Welch’s correction for unequal variance.
