# Supplementary material for: RIPK3 controls MAIT cell accumulation during development but not during infection
Source: Cell Death Dis. 2023 Feb 11;14(2):111. doi: 10.1038/s41419-023-05619-0 (PMC9922319; doi:10.1038/s41419-023-05619-0)
Supplement: Supplementary file 2 — Full length Western Blots [file 41419_2023_5619_MOESM2_ESM.pdf]

A

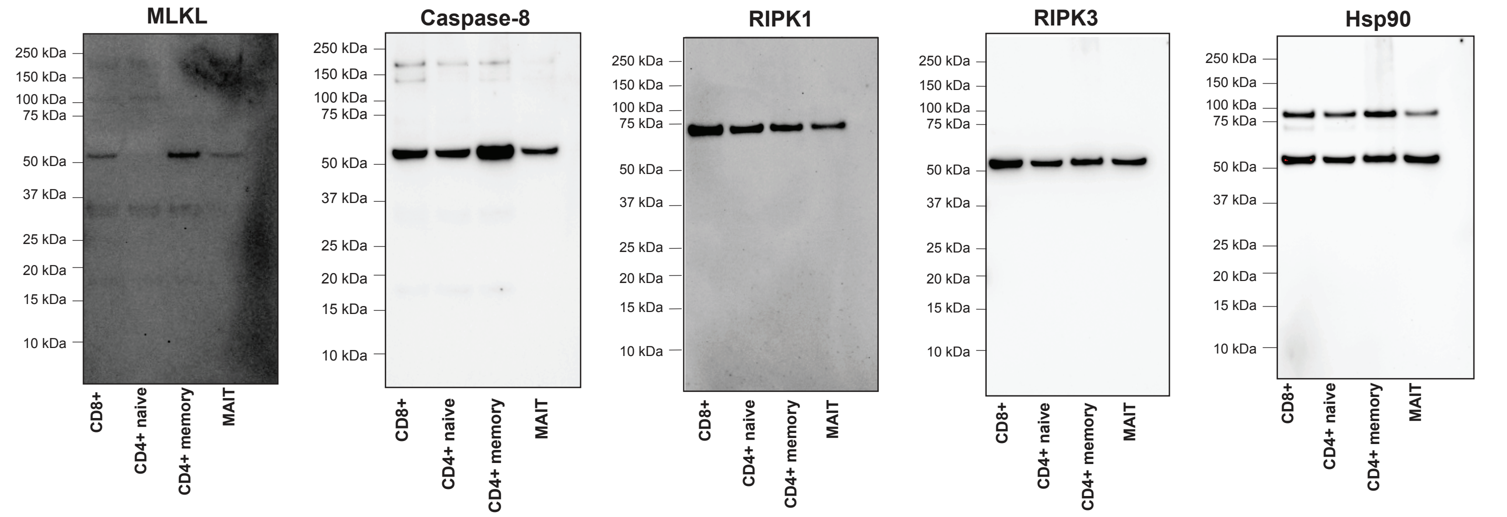

B

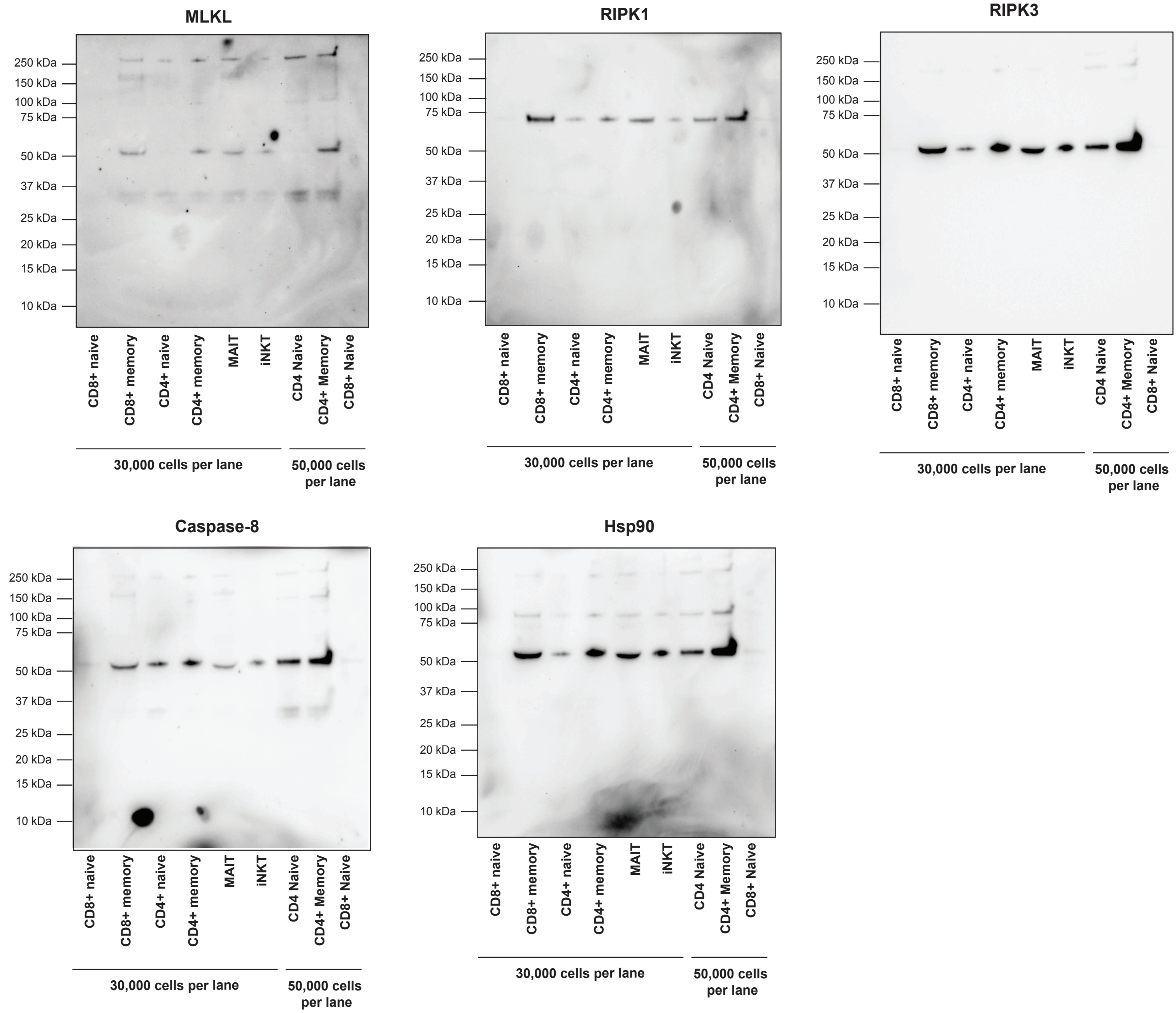

(A) Full Western blots shown from cropped images in Fig 1A, showing MLKL, Caspase-8, RIPK1, RIPK3 and the Hsp90 house keeping control. Lanes are loaded (left to right) with $5x10^4$ FACS CD8+ conventional T cells, CD4+ naïve conventional T cell, CD4+ memory conventional T cells and MAIT cells FACS sorted from the liver.

(B) Full Western blots shown from cropped images in Fig S2A, with lanes loaded left to right with the indicated concentration of CD8+ naïve, CD8+ memory, CD4+ naïve and CD4+ memory conventional T cells, MAIT cells and iNKT cells; each FACS sorted from 12 pooled spleens.

C

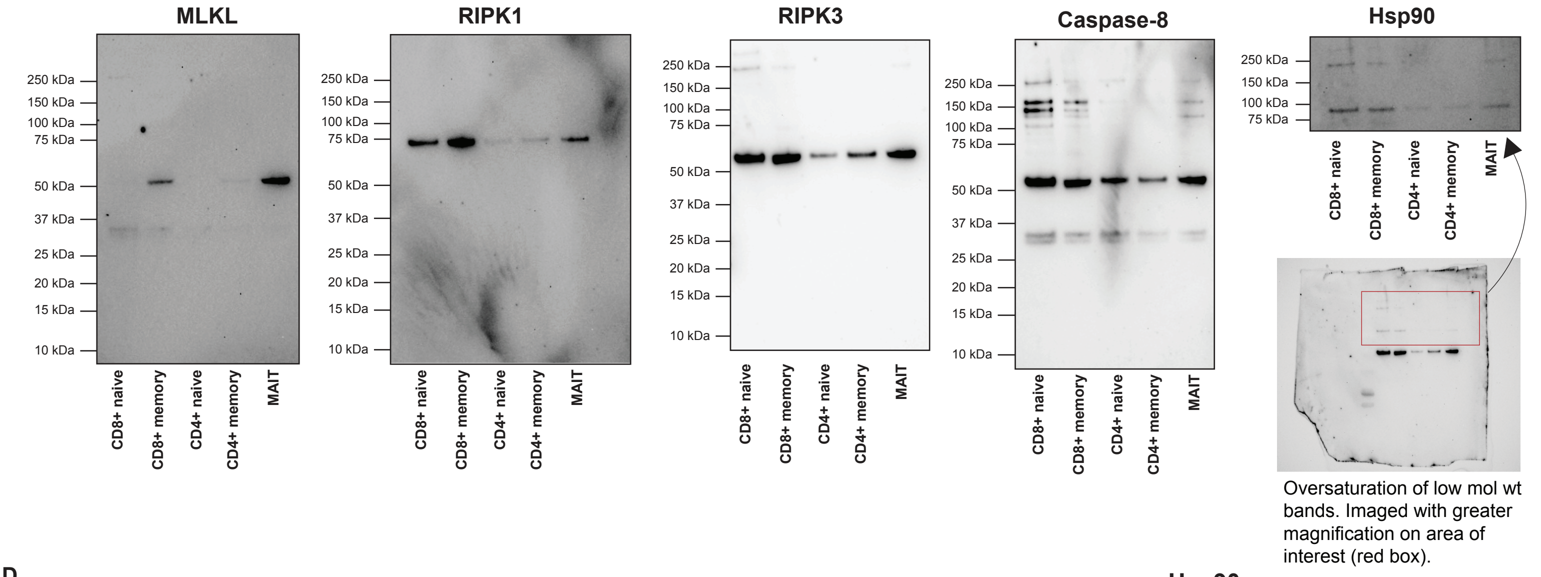

D

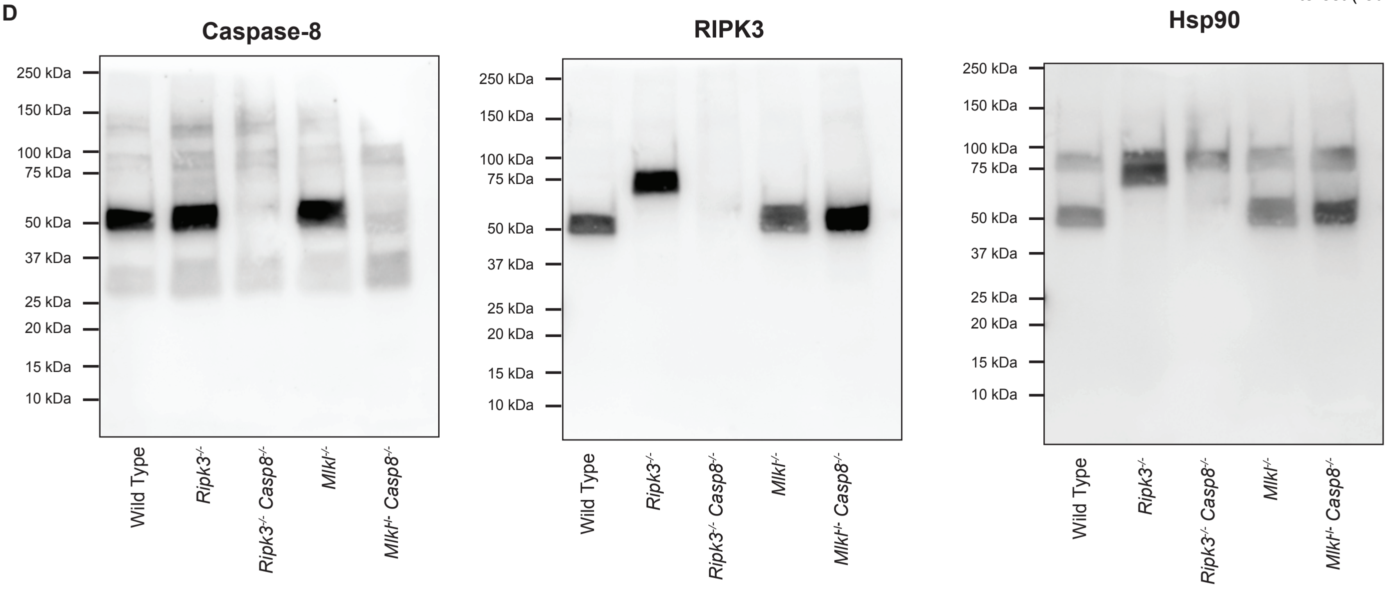

E

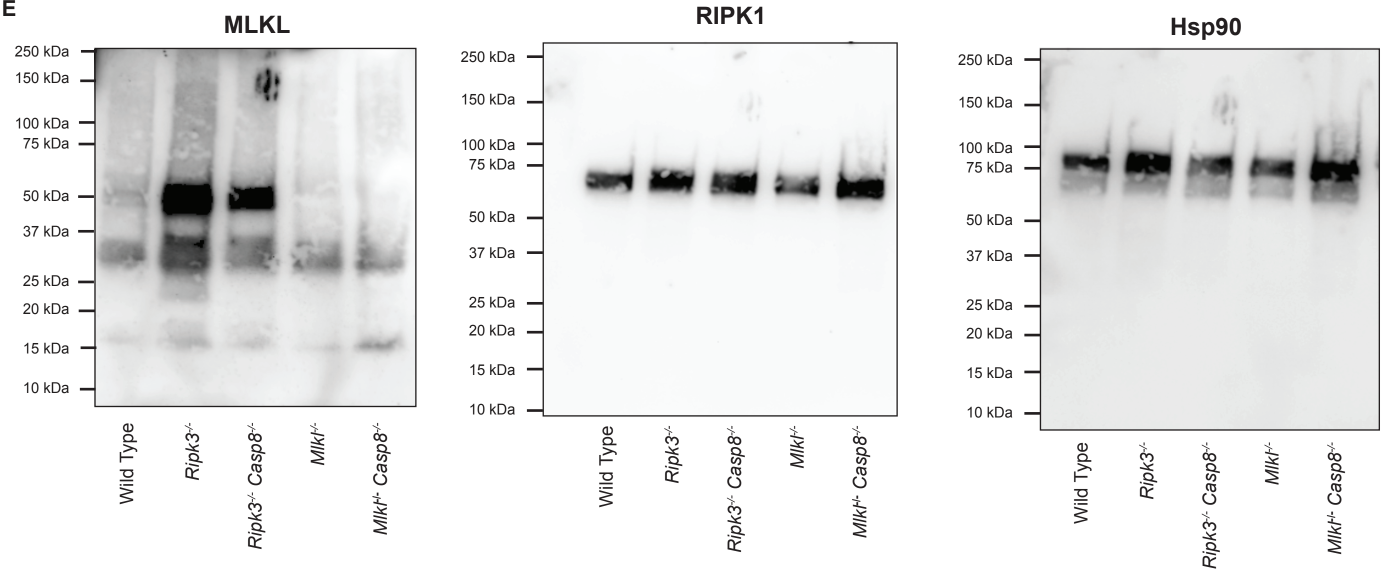

(C) Full Western blots shown from cropped images in Fig S2B, with lanes loaded (left to right) with $5x10^4$ CD8+ naïve, CD8+ memory, CD4+ naïve and CD4+ memory conventional T cells, and MAIT cells; each FACS sorted from spleens pooled from four mice four weeks after MAIT cell boosting with 5-OP-RU and CpG as previously described (Zhao Z, Wang H, Shi M, Zhu T, Pediongco T, Lim XY, et al. Nat Commun. 2021;12[1]:4355).
(D) Full western blots shown from cropped images in Fig S4A, with lanes loaded (left to right) with $2x10^5$ B220-TCRb+ FACS sorted splenocytes from Wild type, *Ripk3*$^{-/-}$, *Ripk3*$^{-/-}$*Casp8*$^{-/-}$, *Mlkl*$^{-/-}$ and *Mlkl*$^{-/-}$*Casp8*$^{-/-}$ mice.
(E) Full western blots shown from cropped images in Fig S4B, with lanes loaded (left to right) with $2x10^5$ B220-TCRb+ FACS sorted splenocytes from Wild type, *Ripk3*$^{-/-}$, *Ripk3*$^{-/-}$*Casp8*$^{-/-}$, *Mlkl*$^{-/-}$ and *Mlkl*$^{-/-}$*Casp8*$^{-/-}$ mice.
